# Aromatic amino acid metabolites alter interferon signaling and influenza pathogenesis

**DOI:** 10.3389/fmolb.2023.1232573

**Published:** 2024-01-23

**Authors:** Gautam Anand, Colin Clark-Dinovo, Alexandra M. Perry, Victoria M. Goodwin, Emma St. Raymond, Sonia Sakleshpur, Ashley L. Steed

**Affiliations:** Department of Pediatrics, Washington University School of Medicine, Saint Louis, MO, United States

**Keywords:** microbiota, microbial metabolites, interferon, inflammation, influenza

## Abstract

The ability of gut microbial metabolites to influence the host is increasingly recognized. The microbiota extensively metabolizes the three aromatic amino acids, tryptophan, tyrosine, and phenylalanine. Previously we have found that a metabolite of tyrosine, 4-OH-phenylpropionic acid, can enhance type I interferon (IFN) signaling and protect from influenza pathogenesis in a murine model. Herein we screened 17 related aromatic amino acid metabolites for effects on IFN signaling in human lung epithelial cells and monocytes alone and in the presence of IFN-β, influenza, and LPS. While the tryptophan family metabolites reduced IFN signaling in both cell types, the tyrosine and phenylalanine metabolites had varied effects, which were cell-type dependent. Pooled treatment of all these metabolites reduced IFN signaling in both cell types and suggested a tryptophan metabolite effect dominance. Strikingly, when all the metabolites were pooled together, we found reduced influenza recovery in both cell types. RNA sequencing further validated reduced viral loads and decreased IFN signaling. Single gene silencing of significantly upregulated genes identified by RNA sequencing (*EGR2*, *ATP6VD02*, *SPOCK1*, and *IL31RA*) did not completely abrogate the metabolite induced decrease in IFN signaling. However, these upregulated targets suggested a mechanistic link to TGF-beta signaling. Treatment with a TGF-beta inhibitor and combined targeted gene silencing led to a significant reversal of metabolite induced IFN signaling suppression. Finally, we demonstrated that intranasal administration of these metabolites prior to influenza infection led to reduced animal morbidity, viral titers, and inflammation. Our work implies that microbial metabolites can alter IFN signaling mechanistically through TGF-beta and promote beneficial outcomes during influenza infection.

## 1 Introduction

The gut microbiota affects the host through a myriad of interactions in which microbial metabolites play a crucial role, especially in immune system regulation. The immunomodulatory effects of these metabolites on the gastrointestinal system have been well documented (Agus et al., 2021; Gasaly et al., 2021; Krautkramer et al., 2021). These metabolites also enter the systemic circulation and impact distant organs such as the brain, liver, lungs, and heart (Wikoff et al., 2009; Nicholson et al., 2012; Schroeder and Backhed, 2016; Liu et al., 2020). Recent findings have identified the microbiota and microbial metabolites as critical modulators of the host immune response, including that to distant viral infections such as influenza (Steed et al., 2017; Trompette et al., 2018; Kumar et al., 2021; Laursen et al., 2021). These metabolites can reach systemic concentrations at or even in excess (0.01–104 µM) of typical drug doses (Schulz et al., 2012; Dodd et al., 2017; Liu et al., 2020). However, the mechanisms by which these metabolites shape the host immune response remain unclear. The aromatic amino acids phenylalanine, tyrosine, and tryptophan are among the crucial building blocks for host physiology and their catabolism by the microbiota yields numerous metabolites that affect the host immune system and response to pathogens (Danaceau et al., 2003; Cervenka et al., 2017; Roager and Licht, 2018). Specifically, aromatic amino acid metabolites have been demonstrated to be both antiviral and anti-inflammatory (Steed et al., 2017).

In this study, we sought to determine the effect of the aromatic amino acids and their metabolized derivatives on IFN signaling and influenza pathogenesis. Specifically, we investigated phenylalanine and its family members, herein referred to collectively as F with its members as follows: phenylalanine, phenylpyruvic acid, phenylacetic acid, phenyllactic acid, phenylacrylic acid, and phenylpropionic acid; tyrosine and its family members, herein referred to collectively as Y with its members as follows: tyrosine, 4-OH-phenylpuruvic acid, 4-OH-phenylacetic acid, 4-OH-phenyllactic acid, 4-OH-phenylacrylic acid, and 4-OH-phenylpropionic acid; and tryptophan and its family members, herein referred to collectively as W with its members as follows: tryptophan, indolepyruvic acid, indoleacetic acid, indolelactic acid, indoleacrylic acid, and indolepropionic acid. These metabolites are increasingly recognized as key mediators in host-microbial interactions, and their microbial producers as well as their known biological activities and host concentrations when characterized have been reviewed extensively (Dodd et al., 2017; Liu et al., 2020).

We hypothesized these single aromatic amino acid metabolites would have important cell type specific effects on innate immune signaling and host-pathogen outcomes. Our previous work identified that one of the tyrosine metabolites, 4-OH-phenylpropionic acid, protects from influenza through a type 1 IFN dependent mechanism (Steed et al., 2017). Given that these aromatic acid acids are metabolized by similar or same niche microbiota and concurrently produced, we also hypothesized that their effects may be synergistic and the impact of a composition of these metabolites is crucial to understand. Herein, we screened for metabolite-induced alterations in IFN signaling in human alveolar epithelial cells and monocytes and found that IFN signaling was most often suppressed by these metabolites in both cell types. Pooled treatment with amino acid metabolite families (F, Y, or W) or a combination of all these metabolites together (FYW) reduced IFN signaling in both cell types, albeit more so in epithelial cells than monocytes, and showed a dominant effect of tryptophan metabolites. Finally, we demonstrate that these metabolites alter IFN signaling through a TGF-beta dependent mechanism and improve host outcomes upon influenza challenge.

## 2 Materials and Methods

### 2.1 Virus

All experiments were performed with influenza A strain PR8 (A/Puerto Rico/8/1934 (H1N1)).

### 2.2 Mice and *in-vivo* infections

All mice were originally obtained from Jackson Laboratories (Bar Harbor, Maine, United States), subsequently maintained at Washington University School of Medicine under specific pathogen-free conditions and bred in-house. Adult (8–16 week-old male and female) mice were used for all infections. Mice were anesthetized with isoflurane and intranasally administered 500 plaque forming units (pfu) of influenza PR8 strain.

### 2.3 Aromatic amino acid and metabolites

The aromatic amino acids and their derivative metabolites were sourced from Sigma Chemicals (St. Louis, United States). The following list enumerates all the metabolites utilized in this study: L-phenylalanine (catalog number: P8740-100G), phenylpyruvic acid (286958-5G), phenylacetic acid (P16621-5G), phenyllactic acid (P7251-10G), phenylacrylic acid (Trans-cinnamic acid; C80857-5G), phenylpropionic acid (W288918-100G-K), L-tyrosine (93829-25G), 4-OH-phenylpyruvic acid (114286-5G), 4-OH-phenylacetic acid (H50004-5G), 4-OH-phenyllactic acid (H3253-100MG), 4-OH-phenylacrylic acid (8002370010), 4-OH-phenylpropionic acid (H52406-5G), L-tryptophan (T0254-5G), indolepyruvic acid (I7017-1G), indoleacetic acid (I2886-5G), indolelactic acid (I5508-250 MG-A), indoleacrylic acid (I2273-1G), and indolepropionic acid (220027-1G). The solvent used for all metabolite preparations was dimethyl sulfoxide (DMSO). Given the potential toxicity of handling DMSO, all necessary precautions to protect our team included use of personal protective gear with nitrile gloves, safety glasses, and laboratory coats. As special precautions must be taken to avoid inhalation of its vapor and potential for combustion, DMSO was stored in a dry and well-ventilated location and away from any heat source.

### 2.4 Metabolite preparations

The stock solutions of the individual metabolites were prepared in a laminar flow hood at 1 M concentration in DMSO. These stock solutions were aliquoted and stored at −20°C for the duration of the study. Similar to the individual stock solutions, the combination stock solutions were also prepared at 1 M for each metabolite included, and aliquots of these pooled metabolites combinations were stored at −20°C. To avoid any degradation from freeze-thaw cycles, each small aliquot was discarded after thawing it to prepare the experimental media.

Fresh intermediate solutions were prepared from the stock solutions for each experiment by diluting the metabolites or metabolite combinations by a 10-fold factor into DMSO to yield a concentration of 100 mM for each metabolite tested. The working experimental solution was made by diluting the intermediate solutions by 1000-fold into the test media, generating a final 100 µM metabolite concentration for the experimental test. The control for these experiments had an equal amount of DMSO in the test media. In order to minimize the DMSO in the metabolite combinations, the intermediate solution was prepared in test media. The test media for each cell line is described in Section 2.8.

### 2.5 Lung preparation and staining

Lungs were inflated with formalin at the time of animal sacrifice and harvested into formalin-containing conical tubes. The tissue was serially washed with Dulbecco’s Phosphate Buffered Saline (DPBS), 30% ethanol, and 50% ethanol 48 h after harvesting and stored in 70% ethanol until the tissue was processed for histology. Antigen retrieval was performed with Trilogy solution (920P-09; CELL MARQUE, United States) by boiling for 20 min. RNA *in-situ* hybridization for influenza NS1 expression (RNAscope probe-521181; ACDBio, United States) was performed using ACDBio RNAscope 2.5 HD- Assay RED (322360; ACDBio, United States) per the manufacturer’s instructions.

### 2.6 *Ex-vivo* viral infection

Mice were sacrificed and lungs perfused with 350,000 pfu of influenza strain PR8 or DPBS as control. The lungs were then incubated in DMEM (Sigma-Aldrich, United States) with 10% heat inactivated FBS for 2 h, minced, and treated with the pooled aromatic amino acid treatment (herein referred to as FYW) at an effective concentration of 100 µM for each metabolite or DMSO as volume matched control in 6 well-plates and incubated for 24 h. The supernatant from the cells was then plated on IFN reporter A549 cells (adenocarcinoma human alveolar basal epithelial cells-a549-nfis; InvivoGen, United States) in 96 well-plates (cells seeded at 0.5 × 10^5^ cells per well) for 48 h and read for reporter activity using Quanti-Luc media (rep-qlc1, rep-qlc2; InvivoGen, United States). The minced tissue was harvested and stored in −80°C and later processed for viral load by plaque assay.

### 2.7 Primary cell harvest and treatment

We maintained sterility during the animal sacrifice procedure and plating of the cells. All solutions were prepared and procedures performed in a certified laminar flow hood. Surgical instruments were autoclave sterilized before the procedure; otherwise, we used sterile disposable cell culture products. We maintained standard sterile procedures while working in the hood and wore personal protective gear including 2 sets of nitrile gloves, safety glasses, and disposable laboratory gowns. Primary cells were harvested as follows: murine peritoneal macrophages were collected via lavage of the peritoneal cavity with 10 mL DPBS (Sigma-Aldrich, United States) and alveolar macrophages were isolated from the lungs via collection with DPBS 0.7 mL serial flushes. The isolated cells were pelleted at 3400 g for 5 min and then re-suspended in DMEM media with 10% heat inactivated fetal bovine serum (FBS, Sigma-Aldrich, United States). For bone marrow cells, both tibia and femur were dissected from euthanized mice. The end of the bones were trimmed, and bone marrow flushed using syringe containing Hanks’ Balanced Salt Solution and 20 mM Hepes. Cells were centrifuged at room temperature at 700 g for 5 min. Red blood cell (RBC) lysis was performed using 0.2% NaCl (6 mL) and mixed gently for 30 s. The reaction was stopped by adding equal volume 1.6% NaCl. The cells were filtered through a 70 μm cell strainer and centrifuged at 700 g for 5 min at room temperature. Supernatant was discarded, and the pellet was re-suspended in 1 mL Dulbecco’s Phosphate Buffered Saline prior to counting. Cells were seeded at 0.5 × 10^5^ cells per 96-well and incubated with pooled aromatic amino acids or equal volume of DMSO as control for 48 h and infected with influenza at a multiplicity of infection of 1 (MOI 1) for 24 h.

### 2.8 qRT-PCR

Total mRNA was isolated from A549 and THP1 cells using RNeasy^®^ plus Mini Kit (74104; Qiagen, Germany) as per manufacturer’s instructions. The cDNA was transcribed from 1 ug of mRNA using iScript cDNA synthesis kit (1708890; BIO-RAD, United States). The qPCR reactions were performed in triplicates using qPCR specific primers (Supplementary Table S1) using TB green qPCR premix (639676; TaKaRa, Japan) on a CFX96 Touch Real-Time PCR Detection System (BIO-RAD, United States). The fold change expression (-ΔΔCt) was calculated after normalization with β2-microglobulin (β2M) expression.

### 2.9 Cell lines and test media

A549-Dual (adenocarcinoma human alveolar basal epithelial cells-a549-nfis; InvivoGen, United States) and THP1-Dual (human lung monocytes; InvivoGen, United States) cells stably express an interferon regulatory factor-inducible Lucia luciferase reporter construct. A549-Dual growth media was DMEM supplemented with 10% heat-inactivated FBS, 1% v/v of penicillin/streptomycin, 100 μg/mL Normocin/Zeocin (InvivoGen, United States), and 100 μg/mL Blasticidin (InvivoGen, United States). THP1-Dual cells were cultured in RPMI 1640 (Sigma-Aldrich, United States) medium supplemented with 10% heat-inactivated FBS, 2 mM L-glutamine, 25 mM HEPES (Sigma-Aldrich, United States), 1% v/v of penicillin/streptomycin, 100 μg/mL Normocin/Zeocin, and 100 μg/mL Blasticidin. The test media for A549 and THP1 cells excluded Zeocin and Blasticidin from their respective growth media. Of note, DMEM and RPMI at baseline contain known quantities of phenylalanine, tryptophan, and tyrosine; these metabolites are quantified by their respective manufacturers as 400 µM phenylalanine, 78 µM tryptophan, and 462 µM tyrosine for DMEM and 91 µM phenylalanine, 25 µM tryptophan, and 111 µM tyrosine for RPMI. Data is not available for the additional amino acid derivatives tested herein. Furthermore, the addition of FBS also contains variable concentrations of these amino acids and their derivatives, which likely vary by batch. Therefore each experiment is appropriately controlled with test media (DMEM or RPMI) with same batch FBS without the tested amino acid metabolite(s) to equalize their baseline concentrations in the media and FBS.

### 2.10 Reporter cell luciferase measurement

Luciferase reporter A549-Dual or THP1-Dual cells were seeded at 0.5 × 10^6^ or 0.5 × 10^5^ cells per well in 6-well or 96-well plates, respectively, and treated per experimental conditions described (for example, single metabolite or pooled metabolites diluted into media on test cells). Twenty-four hours later, 20 μL of the above cell culture supernatant was transferred to another 96-well plate and mixed with 50 μL of QUANTI-Luc and immediately read on a luminometer plate reader (Infinite M200 Pro, TECAN Life Sciences, Switzerland) at a 0.1 s read time to quantify luciferase activity according to manufacturer’s instructions.

### 2.11 Plaque assay

To assess lung viral titers, lung tissue was collected in 1 mL of DPBS and subject to homogenization in Qiagen Tissue LyserLT (Qiagen, United States) using Lysing Matric C tubes with beads (116912050-CF; MPBiomedicals, United States). Plaque assays was performed on MCDK cells with MEM overlay containing 1% oxoid agar, 0.125% BSA (SRE0036; Sigma, United States) and 2 μg N acetylated trypsin (T6763; Sigma, United States). Plaques were visualized by crystal violet. The limit of detection of the influenza plaque assays was 50 pfu/lung tissue or 50 pfu/mL cell supernatant.

### 2.12 RNA sequencing

Agilent Bioanalyzer or 4200 Tapestation was utilized for analyzing total RNA integrity. Total RNA (5–10 μg) with a Bioanalyzer RIN score of greater than 8.0 was used for library preparation. Removal of ribosomal RNA was done by poly-A selection utilizing Oligo-dT beads (mRNA Direct kit, Life Technologies). Fragmentation of mRNA was done in reverse transcriptase buffer by heating to 94°C for 8 min cDNA preparation was done using random hexamers and SuperScript III RT enzyme (Life Technologies, per manufacturer’s instructions). DS-cDNA was prepared by a second-strand reaction. The cDNA thus prepared was blunt ended and had 3′ ends with A base added. The Illumina adapters were then ligated to the ends. Unique dual index tags were introduced into the ligated fragments by specific primers (12–15 cycle amplification). The amplified fragments were then sequenced on an Illumina NovaSeq-6000 using paired end reads extending 150 bases. The adapter sequences are as follows:

**Table:** 

5′ P-GATCGGAAGAGCGGTTCAGCAGGAATGCCGAG
5′ ACA​CTC​TTT​CCT​ACA​CGA​CGC​TCT​TCC​GAT​CT

The primers sequences (X’s indicate index tag) are as follows:

**Table:** 

5′AATGATACGGCGACCACCGAGATCTACACXXXXXXXXXXACACTCTTTCCCTACACGACGCTCTTCCGATCT
5′CAAGCAGAAGACGGCATACGAGAT**XXXXXXXXXX**GTGACTGGAGTTCAGACGTGTGCTCTTCCGA

Library kit manufacturer’s protocol were followed for sample preparation. Illumina’s bcl2fastq software was used for basecalls, and demultiplexing was done using a custom python demultiplexing program with no more than one mismatch in the indexing read. STAR version 2.5.1a was used to align RNA-seq reads to the Ensembl release 76 primary assembly (Dobin et al., 2013). Gene counts were derived from the number of uniquely aligned unambiguous reads by Subread:featureCount version 1.4.6-p5 (Liao et al., 2014). Known Ensembl transcripts isoform expression was estimated with Salmon version 0.8.2 (Patro et al., 2017). The sequencing performance was analyzed for features detected, total number of aligned reads, and total number of uniquely aligned reads. The ribosomal fraction, known junction saturation, and read distribution over known gene models were quantified with RSeQC version 2.6.2 (Wang et al., 2012).

Gene counts were imported into EdgeR (R/Bioconductor package) (Robinson et al., 2010). To adjust for samples with differences in library size, TMM normalization size factor was calculated. For further analysis, samples greater than one-count-per-million were excluded as well and ribosomal genes and genes not expressed in the smallest group minus one. The TMM size factors and the matrix of counts were then imported into the R/Bioconductor package Limma (Ritchie et al., 2015). Observed mean-variance of every gene and sample was used to calculate the Weighted likelihoods with the voomWithQualityWeights (Liu et al., 2015). Resident standard deviation plots of every gene to their average log-counts (with a robustly fitted trend line of the residuals) was used to assess the performance of all genes. Differential gene expression analysis was performed for differences between conditions, and the results were filtered for only those genes with a Benjamini–Hochberg false-discovery rate adjusted *p*-values less than or equal to 0.05.

For each contrast extracted with Limma, MSigDb global perturbations in known Gene Ontology (GO) terms and KEGG pathways were detected using the R/Bioconductor package GAGE (Luo et al., 2009) to test for changes in expression of the log 2 fold-changes reported by Limma in each term versus the background log 2 fold-changes of all genes found outside the respective term. The R/Bioconductor package heatmap3 (Zhao et al., 2014) was used to generate heatmaps across groups of samples for each GO or MSigDb term with a Benjamini–Hochberg false-discovery rate adjusted *p*-value ≤ 0.05. Perturbed KEGG pathways where the observed log 2 fold-changes of genes within the term were significantly perturbed in a single-direction versus background or in any direction compared to other genes within a given term with *p*-values ≤ 0.05 were rendered as annotated KEGG graphs with the R/Bioconductor package Pathview (Luo and Brouwer, 2013).

To find the most critical genes, the raw counts were variance stabilized with the R/Bioconductor package DESeq2 (Love et al., 2014) and was then analyzed via weighted gene correlation network analysis with the R/Bioconductor package WGCNA (Langfelder and Horvath, 2008). Briefly, all genes were correlated across each other by Pearson correlations and clustered by expression similarity into unsigned modules using a power threshold empirically determined from the data. An eigengene was then created for each *de novo* cluster, and its expression profile was then correlated across all coefficients of the model matrix. As these clusters of genes were generated by expression profile rather than their known functional similarity, clustered modules were assigned with the names of random colors wherein grey being the only module that has any pre-existing definition of containing genes that do not cluster well with others. These *de novo* clustered genes were further tested for functional enrichment of known GO terms with hypergeometric tests available in the R/Bioconductor package clusterProfiler13. Significant terms with Benjamini–Hochberg adjusted *p*-values < 0.05 were then collapsed by similarity into cluster. Profiler category network plots were used to depict the most significant terms for each module of hub genes with the aim to interpolate the function of each significant module. The data for all clustered genes for every module were then combined with their respective statistical significance results from Limma to ascertain whether those features were also found to be significantly differentially expressed or not. The data was then analyzed using Reactome for pathway analysis.

### 2.13 siRNA experiments and TGF-B1 inhibition

siRNA experiments utilized the following RNAs: Spock1-s224676; EGR2-s4542; IL31RA-s43773; ATP6VD02-s48333, control-4390843 (Invitrogen, United States). Transfection was performed with Lipofectamine RNAiMAX (13778-150; Invitrogen, United States). For TGF-β1 inhibition, TGF-β inhibitor (SB431542; Selleck chem, United States) was used according to the manufacturer’s instructions (10 µM).

### 2.14 Statistics

GraphPad Prism (San Diego, CA, United States) version 10.1.0 software was used to perform all statistical analyses as described.

### 2.15 Study approval

All mice experiments were performed according to the approved protocol (#20190768) by the Washington University in St. Louis School of Medicine.

## 3 Results

### 3.1 Aromatic amino acid metabolites influence IFN signaling in lung epithelial cells and monocytes

**FIGURE 1:**
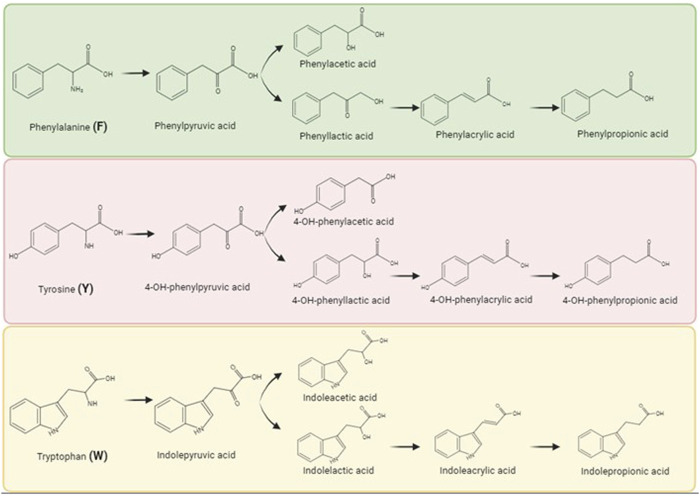
Aromatic amino acids and their metabolites as produced by biochemical breakdown by gut microbiota. Commensal bacteria generates metabolites by metabolically breaking the parent aromatic amino acids, namely, phenylalanine (F), tyrosine (Y), and tryptophan (W). Biorender was used to make this figure (https://www.biorender.com/).

Given that microbial metabolites can influence host systemic immunity, we sought to understand if these metabolites had cell specific effects. Our prior work has demonstrated that one aromatic amino acid metabolite, 4-OH-phenylpropionic acid, enhanced IFN signaling (Steed et al., 2017) and therefore we chose this signaling pathway for further investigation with a range of sequential metabolites of phenylalanine, tyrosine, and tryptophan (Figure 1). We hypothesized that the impact of the aromatic amino acid metabolites on IFN signaling would be cell type specific. Given that the physiologic concentrations of these metabolites are largely unknown or incomplete, we chose to increase the metabolite dose by 100 μM for each metabolite given that the pooled metabolites each at 100 μM concentration increase exhibited no cell cytotoxicity (Supplementary Figure S1) as well as prior studies demonstrating immunomodulatory effects at these doses. Dodd et al. demonstrated that *C. sporogenes* can generate aromatic amino acid concentrations up to 600 μM in bacterial cell culture supernatants and *in vivo* monoclonization with *C. sporogenes* led to serum concentrations of these indoles of 80 μM (Elsden et al., 1976; Smith and Macfarlane, 1996; Danaceau et al., 2003; Russell et al., 2013; Dodd et al., 2017; Steed et al., 2017; Liu et al., 2020).

We screened 17 aromatic amino acid metabolite derivatives for their effect on IFN signaling in 2 human reporter cells lines, A549 lung epithelial cells and THP1 monocytes, given our interest in IFN modulation during influenza pathogenesis. We tested the individual metabolites’ effect on IFN signaling alone and in the combined presence of various stimuli, namely, IFN-β, influenza, and LPS. For our experiments, it is important to note that the cell culture media contains defined baseline known quantities of phenylalanine, tryptophan, and tyrosine while that in FBS is likely variable by batch. Thus our addition of metabolites increases the final concentration of aromatic amino metabolites by 100 μM and is not definitive of the final concentration. Therefore each experiment was controlled with test media and same batch FBS to equalize their baseline concentrations across control and experimental conditions. While our final concentration may exceed physiologic concentrations for some metabolites, we aimed to investigate if these metabolites could be useful therapeutically in excess.

**FIGURE 2:**
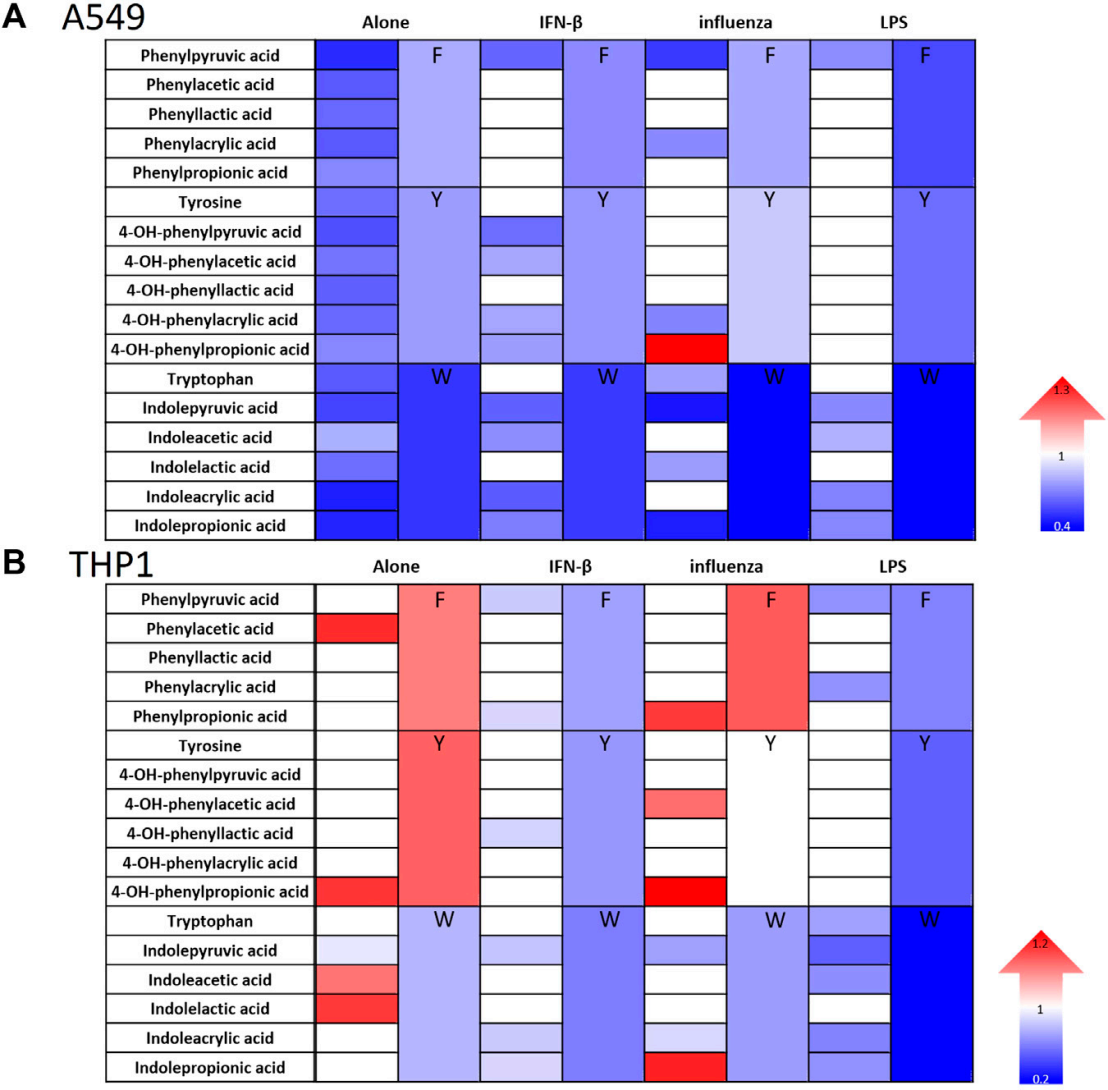
Aromatic amino acid metabolites modulate IFN signaling in human lung epithelial cells and monocytes. Heatmaps depicting fold change in IFN reporter activity in A549 **(A)** and THP1 cells **(B)** treated with airomatic amino acid metabolites (numbered 2–18) individually and in family combinations (F, Y, W) alone and with IFN-β (10 U/mL), influenza (MOI-1) or LPS (5 ng/mL) for 24 h. DMSO served as the control for the metabolites. (*n* = 5 experiments: 23 biological and 23 technical replicates for A549 individual metabolites alone and *n* = 3 experiments: 11 biological and 11 technical replicates for THP1 individual metabolites alone; *n* = 3 experiments: 11 biological and 11 technical replicates for A549 and THP1 with IFN-β and individual metabolites; *n* = 3 experiments: 11–15 biological and technical replicates for A549 with IFN-β and *n* = 3 experiments: 11–15 biological and technical replicates for THP1 with influenza and individual metabolites; *n* = 3 experiments: 11 biological and 11 technical replicates for A549 and THP1 with LPS and individual metabolites; *n* = 3 experiments: 6–24 biological and technical replicates for A549 alone with metabolite family combinations; *n* = 3 experiments: 12–20 biological and technical replicates for THP1 alone with metabolite family combinations; *n* = 3 experiments: 15 biological and technical replicates for A549 with IFN-, 14 biological and technical replicates for A549 with influenza, 12 biological and technical replicates for A549 with LPS and metabolite family combinations; *n* = 3 experiments: 9 biological and technical replicates for THP1 with IFN-β, influenza or LPS along with all metabolite family combinations). Mann–Whitney was used for statistical analysis. If a color is used, the comparison met statistical significance at *p* < 0.05; please see Supplementary Figures S2–S5 for significance magnitude.

Consistent with our prior work in fibroblasts (Steed et al., 2017), 4-OH-phenylpropionic acid, enhanced IFN signaling in the setting of influenza in both cell types (Figure 2). However, IFN signaling most often decreased in A549 cells and had mixed effects in THP1 cells (Figure 2; Supplementary Figures S2–S5). Specifically, the tryptophan metabolites generally reduced IFN signaling in both cell types but more potently in A549 cells. Our results demonstrate that indeed microbial metabolites influence IFN signaling in a cell type specific manner. Importantly, we demonstrated that addition of these metabolites did not interfere with our luciferase reporter assay readout (Supplementary Figure S6).

**FIGURE 3:**
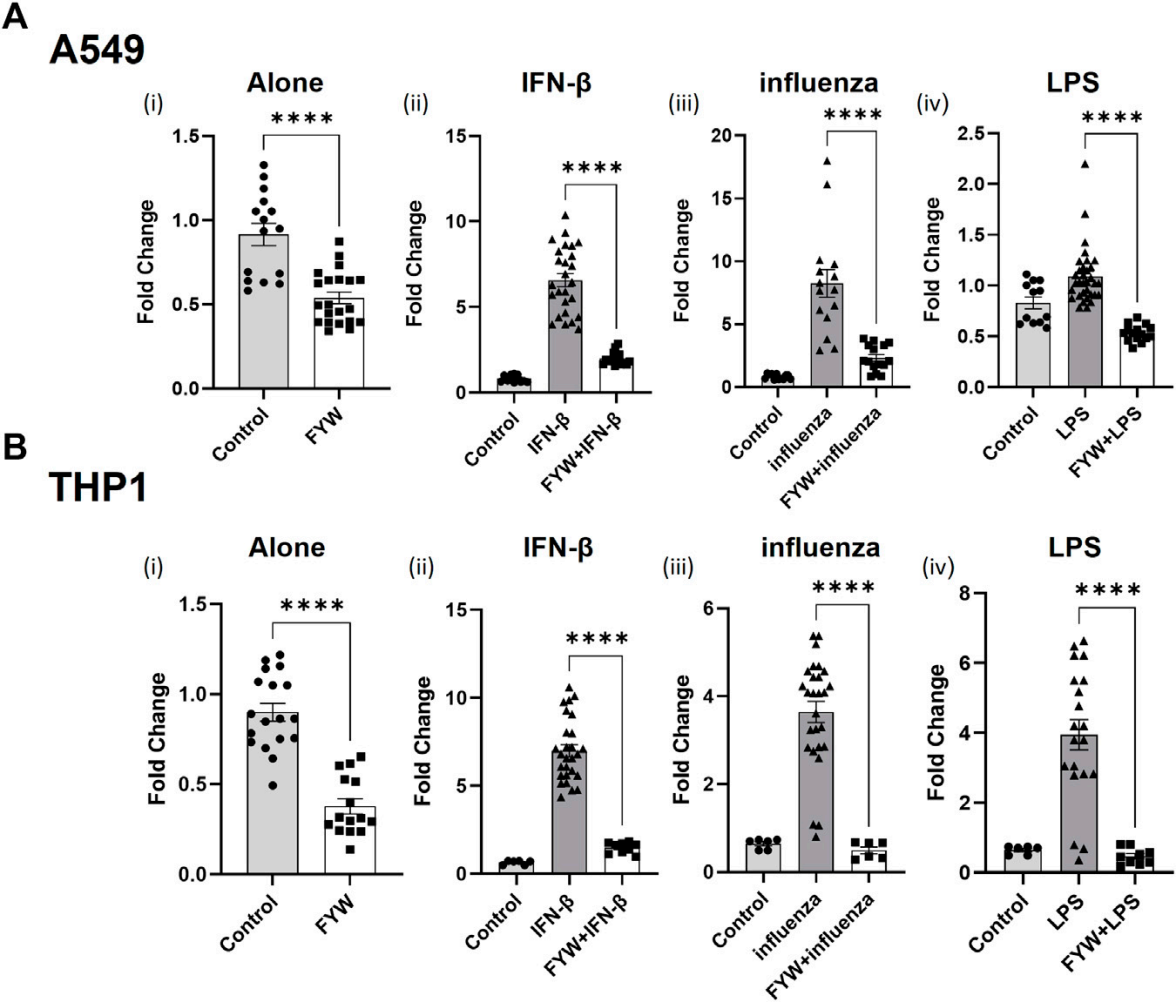
Pooled metabolites of phenylalanine, tyrosine, and tryptophan reduce IFN signaling. Fold change in IFN reporter activities in A549 **(A)** and THP1 **(B)** cells with pooled aromatic amino acid metabolites (FYW) alone **(i)**, and with IFN-β (10 U/mL) **(ii)**, influenza (MOI-1) **(iii)** and LPS (5 ng/mL) **(iv)** for 24 h. FYW treatment includes all metabolites previously numbered 2–18. DMSO served as the control for the metabolites. (*n* = 4 experiments: 15–21 biological and technical replicates for A549 with FYW alone; 12–26 biological and technical replicates for A549 with IFN-β and FYW; 12–33 biological and technical replicates for A549 with influenza and FYW; l2 biological and technical replicates for A549 witlh LPS and FYW; *n* = 4 experiments: 15–18 biological and technical replicates for THP1 with FYW alone; 6–27 biological and technical replicates for THP1 with IFN-β and FYW; 6–27 biological and technical replicates for IFN-β with influenza and FYW; 6–20 biological and technical replicates for THP1 with LPS and FYW). Graphs depict average SEM. ****p < 0.0001. Mann–Whitney was used for statistical analysis.

Based on these findings, we investigated the concomitant effects of these metabolites as they likely exist in combination *in vivo*. We made combinations of these metabolites with their respective amino acid families, namely, phenylalanine family (F), tyrosine family (Y), and tryptophan family (W) as well as a combination of all the metabolites (FYW). While each family of amino acids decreased IFN signaling in the presence of additional stimuli with the exception of Y on monocytes infected with influenza, treatment of both cell types with FYW demonstrated the most pronounced decrease in IFN signaling (Figure 3 and S7). FYW treatment significantly reduced IFN signaling by 2-fold in the absence of other stimuli, 3.4-fold with IFN-β, 3.6-fold with influenza, and 2-fold with LPS in lung epithelial cells (Figure 3A). The FYW treatment also led to similar results in monocytes with IFN reductions of 2.4-fold in the absence of other stimuli, 4.7-fold with IFN-β, 7.4-fold with influenza, and 8.5-fold with LPS (Figure 3B). This effect was dose-dependent over a range of concentrations and sustained over time (Supplementary Figure S8). Importantly, no cytotoxicity was observed in any cells with FYW treatment at the dose and time point chosen for further initial investigation (added 100 μM, 24 h of treatment) (Supplementary Figures S1, S8).

**FIGURE 4:**
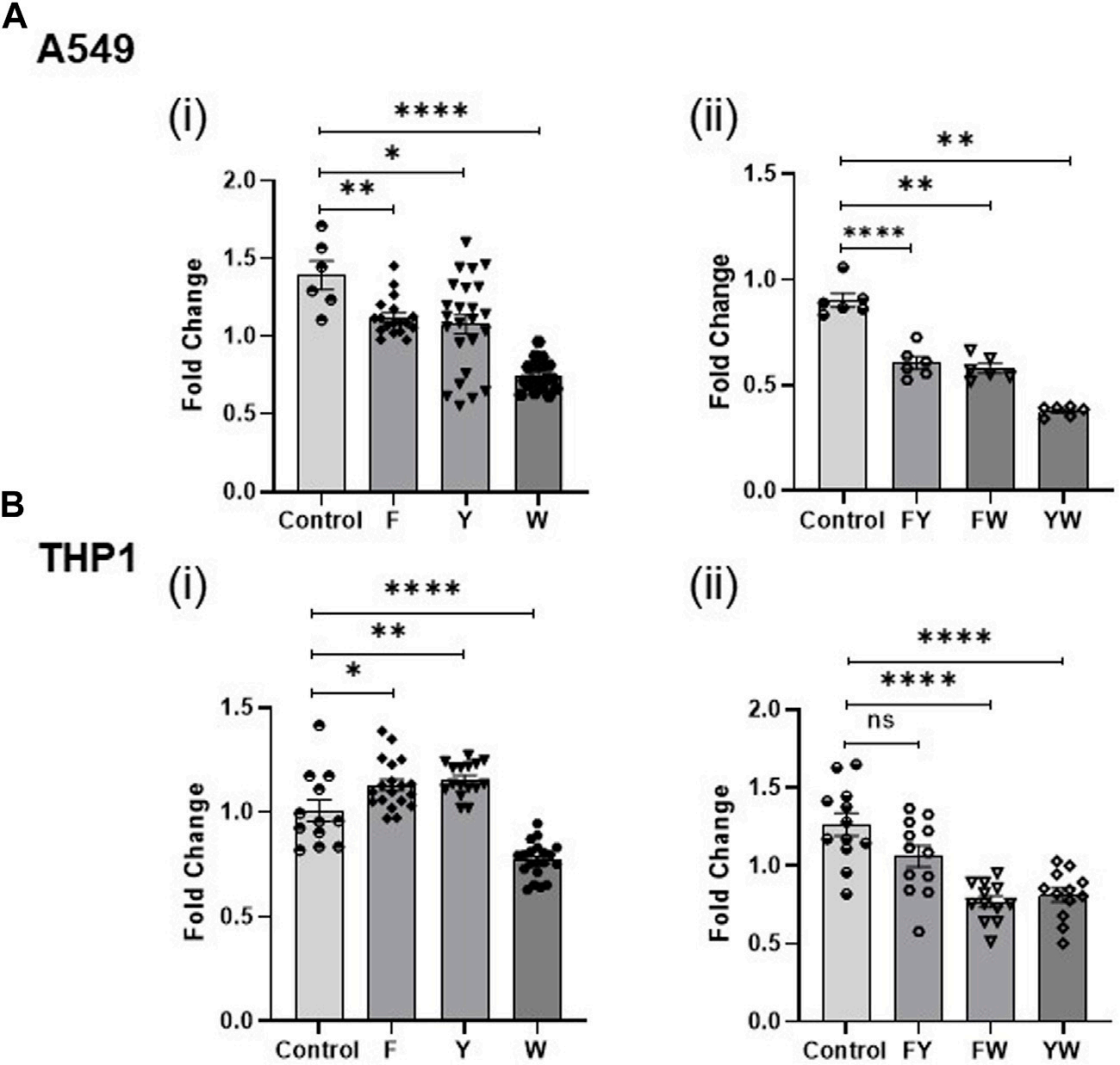
Tryptophan family metabolites drive the reduction in IFN signaling. Fold change in IFN reporter activity in A549 **(A)** and THP1 **(B)** cells treated with pooled aromatic amino acid family metabolite combinations F, Y, and W **(i)** and FY, FW, and YW **(ii)**. DMSO (volume matched) served as the control for the metabolites. (*n* = 3 experiments: 6–24 biological and technical replicates for A549 alone with metabolite family combinations; *n* = 3 experiments: 12–20 biological and technical replicates for THP1 with metabolite family combinations; *n* = 2: 6 biological and technical replicates for A549 witlh two family metabolite combinations; *n* = 2: 12 biological and technical replicates for THP1 with two family metabolite combinations). Graphs depict average SEM. *p < or = 0.05, **p < 0.01, ****p < 0.0001, and ns denotes not statistically significant. Mann–Whitney was used for statistical analysis.

With respect to the individual family combinations, we found that A549 cells had reduced IFN signaling upon treatment with each metabolite family combination (Figure 4i; Supplementary Figures S7i, iii, v). The tryptophan family metabolites exerted the most potent impact on reducing IFN signaling, consistent with our previous observation of its individual metabolites’ effects. THP1 cells responded distinctly from A549 cells and had differential effects on IFN signaling across various conditions (Figure 4Bi; Supplementary Figures S7ii, iv, vi). The phenylalanine family combination (F) lead to elevated IFN signaling when the cells were treated without additional stimuli or with influenza but demonstrated reduced IFN signaling in the presence of IFN-β and LPS. The tyrosine family combination (Y) led to enhanced IFN signaling in the absence of other stimuli but decreased IFN signaling in the presence of LPS and IFN-β. However, the tryptophan family combination (W) consistently led to a reduction in IFN signaling in the presence and absence of other stimuli in both cell types.

### 3.2 Tryptophan family metabolites drive a reduction in IFN signaling

To determine the contribution of the aromatic amino acid metabolite families, we treated the A549 and THP1 cells with different combinations of FYW such that each combination lacked a specific family of metabolites, i.e., YW, FW, and FY. The tryptophan family metabolites significantly attenuate IFN signaling in comparison to the other two families (Figures 4Aii, Bii). Treatment of cells with FY (omitting the tryptophan family metabolites, W) reversed the previously demonstrated decrement in IFN signaling completely in monocytes and partially in lung epithelial cells. This finding is consistent with the potent effect demonstrated by the individual tryptophan metabolites. However, the combined FYW treatment decreased IFN signaling more significantly than the W family alone, indicating that the three-metabolite families act synergistically to reduce IFN signaling.

### 3.3 FYW treatment primes cells to reduce IFN signaling

**FIGURE 5:**
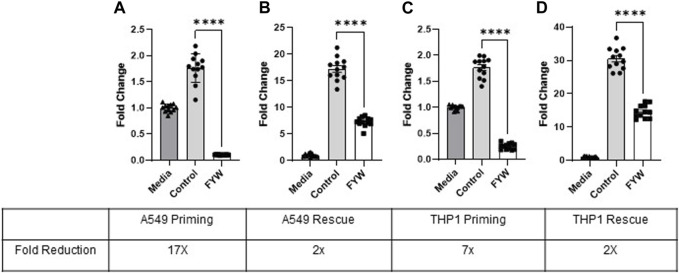
Pretreatment with pooled aromatic amino acid metabolites reduces IFN signaling further than treatment after stimulation. Fold change in IFN reporter activity in A549 **(A)** and THP1 **(C)** cells primed with pooled aromatic amino acid metabolites (FYW) for 48 h and then infected with influenza for 24 h. Fold change in IFN reporter activity in A549 **(B)** and THP1 **(D)** cells infected with influenza for 24 h and then treated with FYW for 24 h. DMSO (volume-matched) served as the control for the metabolites, and media refers to the volume-matched negative control without the DMSO or metabolites. (*n* = 2 experiments; 12 biological and technical replicates for A549 and THP1). Graphs depict average SEM ****p < 0.0001. Mann–Whitney was used for statistical analysis.

FYW led to significantly reduced IFN signaling in both the lung epithelial (A549) cells and monocytes (THP1) when challenged with various stimuli. We next asked whether pretreatment of these cells with FYW prior to additional stimulation (“prime”) would further reduce IFN signaling. Therefore, we treated both cell types with FYW for 48 h before exposure to influenza. We found a dramatic decrease in IFN signaling with a 17-fold and 7-fold reduction in A549 and THP1 cells (Figure 5), respectively. However, when the cells were first exposed to influenza for 24 h and then FYW added for another 24 h (“rescue”), the reduction was effectively lowered although not abolished with an approximate 2-fold decrease in IFN signaling in both A549 and THP1 cells (Figure 5). Murine primary cells also demonstrated similar findings with 2.3-fold, 1.8-fold, and 1.3-fold reduction in IFN signaling, respectively, in isolated alveolar macrophages, bone marrow cells, and peritoneal macrophages (Supplementary Figure S9). These results demonstrate that priming the cells with aromatic amino acid metabolites leads to a potent decrease in IFN signaling in multiple cell types.

### 3.4 FYW treatment leads to decreased viral recovery *in vitro* and *ex vivo*

**FIGURE 6:**
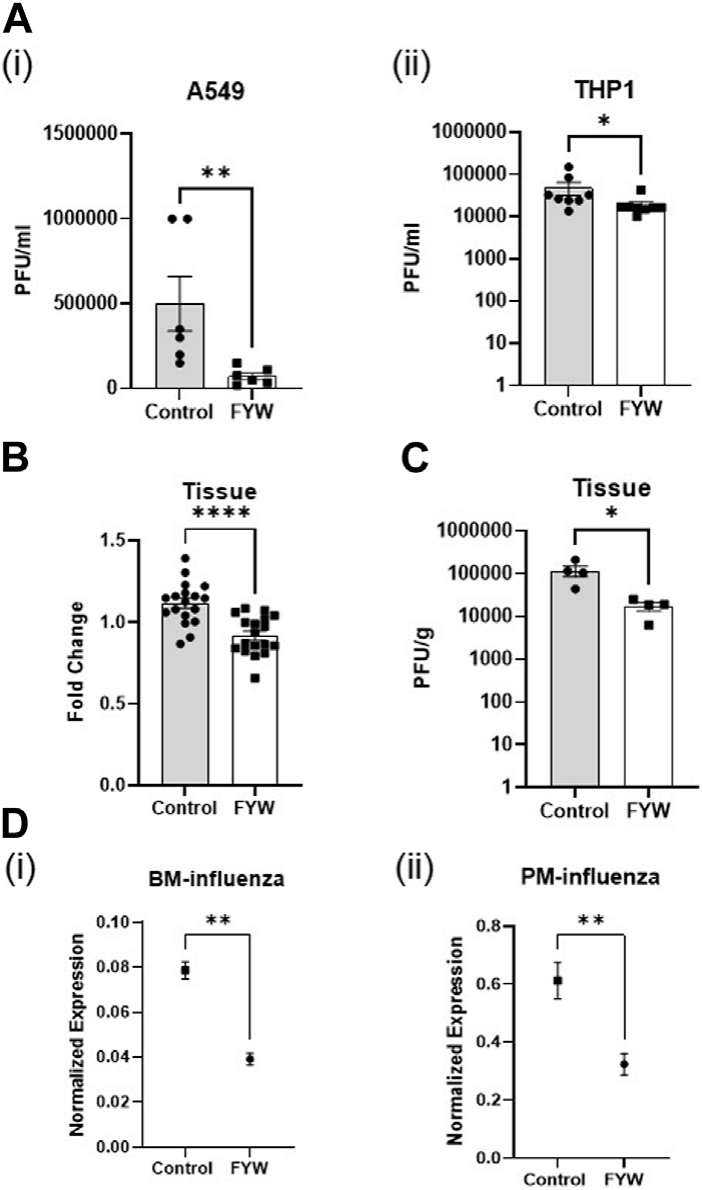
Pooled aromatic amino add metabolites decrease the infectious viral recovery. **(A)** Influenza plaque-forming units recovered from A549 **(i)** and THP1 **(ii)** cells pretreated with FYW for 48 h and then infected with influenza for 24 h. (*n* = 3 experiments; 6 biological and 12 technical replicates for A549 and THP1) **(B)** Fold change in IFN reporter activity in A549 cells at 48 h after treatment with supernatant from *ex-vivo* influenza-infected mouse lungs. (*n* = 2 experiments; 9 biological and 18 technical replicates for A549 and THP1). **(C)** Influenza plaque-forming units recovered from *ex-vivo* infected mouse lungs after treatment with FYW for 24 h. DMSO (volume matched) served as the control for the metabolites. (*n* = 2 experiments; 4 biological and technical replicates). **(D)** RNA expression from real-time PCR for influenza matrix protein in primary bone marrow cells **(i)** and peritoneal macrophages **(ii)** treated with the pooled metabolite combination FYW for 24 h and infected with influenza for 24 h (*n* = 2 experiments: 5 biological and 6 technical replicates for bone marrow cells; 6 biological and technical replicates for peritoneal macrophages). Graphs depict average SEM. *p < or = 0.05, **p < 0.01, ****p < 0.0001. Mann–whitney was used for statistical analysis.

Given that IFN signaling is mechanistically linked to an antiviral cell state, we next investigated the effect of FYW treatment on viral pathogenesis. We primed A549 and THP1 cells with FWY for 48 h and then exposed the cells to influenza (at an MOI of 1). Surprisingly, FYW pretreatment decreased viral recovery after 24 h by 6.8-fold and 2.6-fold in A549 and THP1 cells, respectively (Figure 6A). Next, we elected to use primary cells to more closely mimic natural infection. We lavaged influenza into murine lungs at time of animal sacrifice. After 2 h, we minced the lung tissue and treated with or without FYW. Similar to our cell line results, we found that supernatants from the minced tissue treated with FYW induced less IFN signaling than control (Figure 6B). Again, FYW treatment yielded a marked reduction (6-fold) in viral recovery from the minced lung tissue (Figure 6C). Finally, we isolated murine primary cells, pretreated with FYW treatment for 48 h, and then exposed to influenza for 24 h. Subsequent qRT-PCR analysis for the influenza matrix transcript demonstrated that FYW treatment decreased this viral transcript in both bone marrow cells and peritoneal macrophages (Figure 6D). To test if FYW had a direct negative structural impact on the virus, we performed hemagglutination inhibition assays in the setting of different concentrations of FYW (increased by 50mM to 390 µM). There was no agglutination inhibition with any concentration of FYW tested (Supplementary Figure S10) suggesting that the virus remains structurally intact when exposed to FYW.

### 3.5 FYW treatment leads to a unique transcriptional profile

**FIGURE 7:**
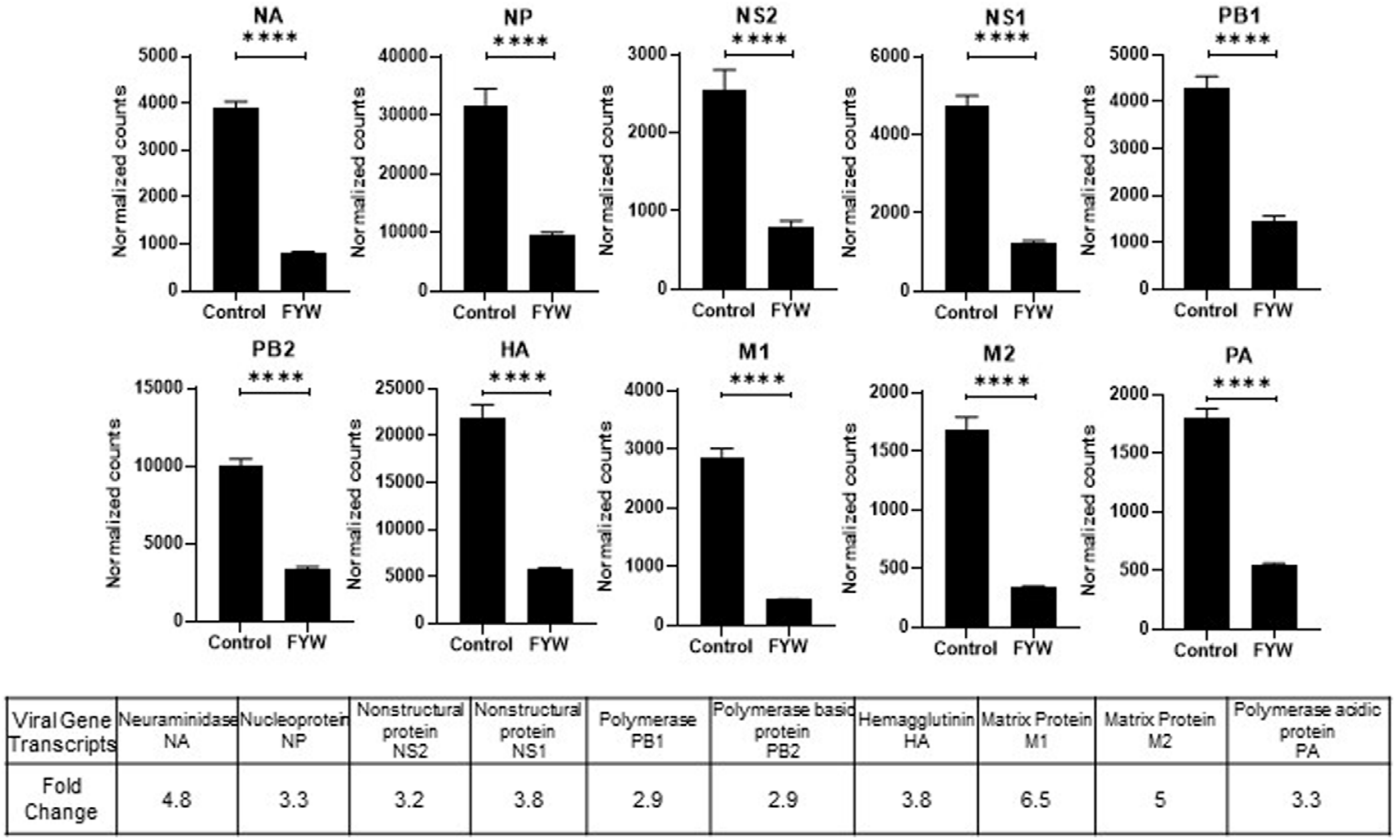
Pooled aromatic amino acid metabolites decrease influenza RNA. **(A)** Viral RNA transcript counts in samples of A549 cells pretreated with FYW for 48 h and then infected with influenza for 24 h. DMSO served as the control for the metabolites. Graphs depict average SEM (*n* = 3 biological replicates). ****p < 0.0001. Mann–Whitney was used for statistical analysis. The bottom table depicts the fold decrease in viral RNA counts graphed above.

**FIGURE 8:**
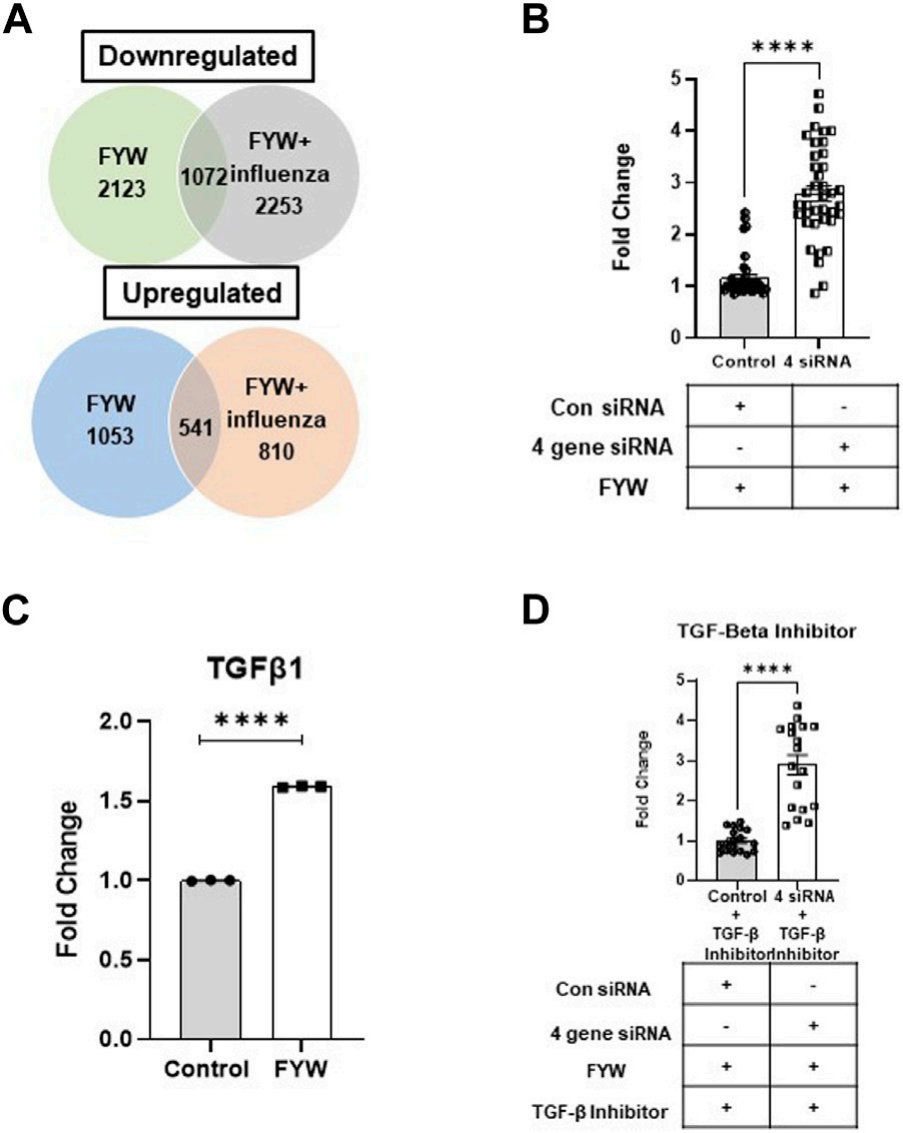
Pooled aromatic amino acid metabolites treatment alters gene expression. **(A)** Venn diagram depicting numbers of differentially expressed genes (DEGs) identified in A549s with pretreatment treatment with FYW for 48 h and then infected with influenza for 24 h. DEGs downregulated and upregulated per the condition depicted compared to DMSO as the control. **(B)** TGF-β1 transcript counts in samples of A549 cells pretreated with FYW for 48 h and then infected with influenza for 24 h. DMSO served as the control for the metabolites. (*n* = 3 biological replicates). **(C)** Fold change in IFN reporter activity in A549 cells transfected with siRNA directed at *spock1*, *egr2*, *il3lra*, and *atp6vd02* or control siRNA for 24 h, and the cells were left to recover for 24 h. The cells were pretreated with FYW for 48 h and infected with influenza for 24 h (*n* = 4 experiments: 18 biological and 38 technical replicates). **(D)** Fold change in IFN reporter activity in A549 cells treated with TGF-β inhibitor for 24 h, then transfected with siRNA directed at *spock1*, *egr2*, *il3lra* and *atp6vd02* or control siRNA for 24 h, and the cells were left to recover for 24 h. The cells were pretreated with FYW for 48 h and infected with influenza for 24 h (*n* = 2 experiments: 9 biological and 18 technical replicates). Graphs depict average SEM. ****p < 0.0001. Mann–Whitney was used for statistical analysis.

Treatment with FYW decreased IFN signaling and viral recovery across a number of cell types. To investigate this seemingly dichotomy further, we determined the transcriptional response to FYW treatment. A549 cells were first primed with FYW or control for 48 h and then exposed or not exposed to influenza (at an MOI of 1) for 24 h. RNA was isolated and analyzed by bulk RNA sequencing. We found reduced viral transcripts across the mapped influenza transcriptome, further verifying that FYW impacts viral replication (Figure 7). In regard to host transcripts, treatment with FYW with or without infection had the greatest number of differentially expressed genes (DEGs) in comparison to control. FYW treatment led to 1053 upregulated DEGs of which 541 were sustained upon infection and 810 unique to FYW pretreatment and infection (Figure 8A). Reactome analysis identified pathways pertaining to cellular responses to stimuli, particularly the endoplasmic reticulum stress and unfolded protein stress responses (Table 1). FYW treatment also revealed 2123 downregulated DEGs. Reactome analysis demonstrated that 25 pathways were downregulated (*p* < 0.01 and FDR <0.05) (Table 2) with IFN signaling and IFN α/β signaling as the most significant (*p* ≤ 1.1e-16 and FDR ≤ 3.43e-14).

**TABLE 1:** Upregulated pathways in response to aromatic amino acid metabolites in A549 alveolar epithelial cells (FDR-False Discovery Rate).

Pathway name	Entities				Reactions	
	Found	Ratio	*p*-value	FDR*	Found	Ratio
Response of EIF2AK1 (HRI) to heme deficiency	19/29	0.002	3.44e-08	6.11e-05	16/20	0.001
ATF4 activates genes in response to endoplasmic reticulum stress	17/34	0.002	6.42e-06	0.005	7/7	5.07e-04
PERK regulates gene expression	19/42	0.003	7.68e-06	0.005	9/11	7.97e-04
Unfolded Protein Response (UPR)	41/155	0.01	5.87e-05	0.026	76/94	0.007
Insulin-like Growth Factor-2 mRNA Binding Proteins (IGF2BPs/IMPs/VICKZs) bind RNA	8/13	8.61e-04	4.68e-04	0.166	3/3	2.17e-04
Type I hemidesmosome assembly	7/11	7.28e-04	8.61e-04	0.193	6/6	4.35e-04
Inhibition of Signaling by Overexpressed EGFR	6/8	5.30e-04	8.71e-04	0.193	2/2	1.45e-04
Signaling by Overexpressed Wild-Type EGFR in Cancer	6/8	5.30e-04	8.71e-04	0.193	2/2	1.45e-04
ATF6 (ATF6-alpha) activates chaperone genes	8/15	9.93e-04	0.001	0.23	5/5	3.62e-04

**TABLE 2:** Downregulated pathways in response to aromatic amino acid metabolites in A549 alveolar epithelial cells (FDR-False Discovery Rate).

Pathway name	Entities				Reactions	
	Found	Ratio	*p*-value	FDR*	Found	Ratio
Interferon Signaling	109/394	0.028	1.11e-16	3.43e-14	66/69	0.005
Interferon alpha/beta signaling	65/186	0.013	1.11e-16	3.43e-14	21/22	0.002
Cell Cycle, Mitotic	154/596	0.042	1.11e-16	3.43e-14	304/350	0.026
Cell Cycle	174/734	0.051	1.11e-16	3.43e-14	368/449	0.033
Cell Cycle Checkpoints	82/280	0.02	1.11e-16	3.43e-14	33/56	0.004
Mitotic Prometaphase	60/211	0.015	4.52e-14	1.16e-11	20/20	0.001
Mitotic G1 Phase and G1/S transition	53/173	0.012	8.37e-14	1.62e-11	86/99	0.007
Amplification of signal from unattached kinetochores via a MAD2 inhibitory signal	38/94	0.007	9.46e-14	1.62e-11	4/4	2.95e-04
Amplification of signal from the kinetochores	38/94	0.007	9.46e-14	1.62e-11	4/4	2.95e-04
M Phase	88/416	0.029	1.02e-12	1.58e-10	71/91	0.007

To probe the mechanism by which FYW reduces IFN signaling, we selected four target genes, Spock1, EGR2, IL31RA, and ATP6VD02, based on those most highly upregulated in our RNA sequencing analysis. qRT-PCR analysis also corroborated the upregulation of these genes upon FYW treatment (Supplementary Figures S1A–iv). Respective siRNAs effectively decreased each transcripts’ expression (Supplementary Figures S11Bv–viii), although we found that the overall the gene expression even after siRNA treatment was still increased by FYW compared to the control. After individual siRNA treatment and then FYW exposure, we found that IFN signaling was still reduced upon influenza exposure, only abrogated by 1.2, 1.3, and 1.4-fold in the case of *EGR2*, IL31RA, and ATP6VD02, respectively (Supplementary Figure S12). We next suppressed gene transcription of all 4 gene targets. After the sequential combined 4 siRNA treatment, FYW priming, and influenza exposure, we found that gene knock-down counteracted the reduction of IFN signaling by 2.8-fold (Figure 8B).

We hypothesized that a central mediator linked to these upregulated DEGs is Transforming Growth Factor-β (TGF-β). This idea was especially intriguing as prior studies have linked reduced IFN-α/β levels with TGF-β signaling (Bedke et al., 2012; Furuya et al., 2015; Denney et al., 2018). Importantly we found an increase in TGF-β in our RNA sequencing results (Figure 8C). To test this hypothesis, we utilized a TGF-β inhibitor that acts by binding to the TGF-β1 receptor. A549 cells were treated with this inhibitor for 24 h and then transfected with all 4 siRNAs for 48 h prior to FYW exposure. After an additional 48 h, the cells were exposed to influenza for 24 h. The transcriptional knock-down in combination with the TGF-β inhibitor led to an alleviation of IFN signaling reduction by another 2.9-fold (Figure 8D). These findings demonstrate that the downregulation of IFN signaling by microbial metabolites is TGF-β dependent.

### 3.6 Microbial metabolites improve outcomes in the setting of influenza pathogenesis

**FIGURE 9:**
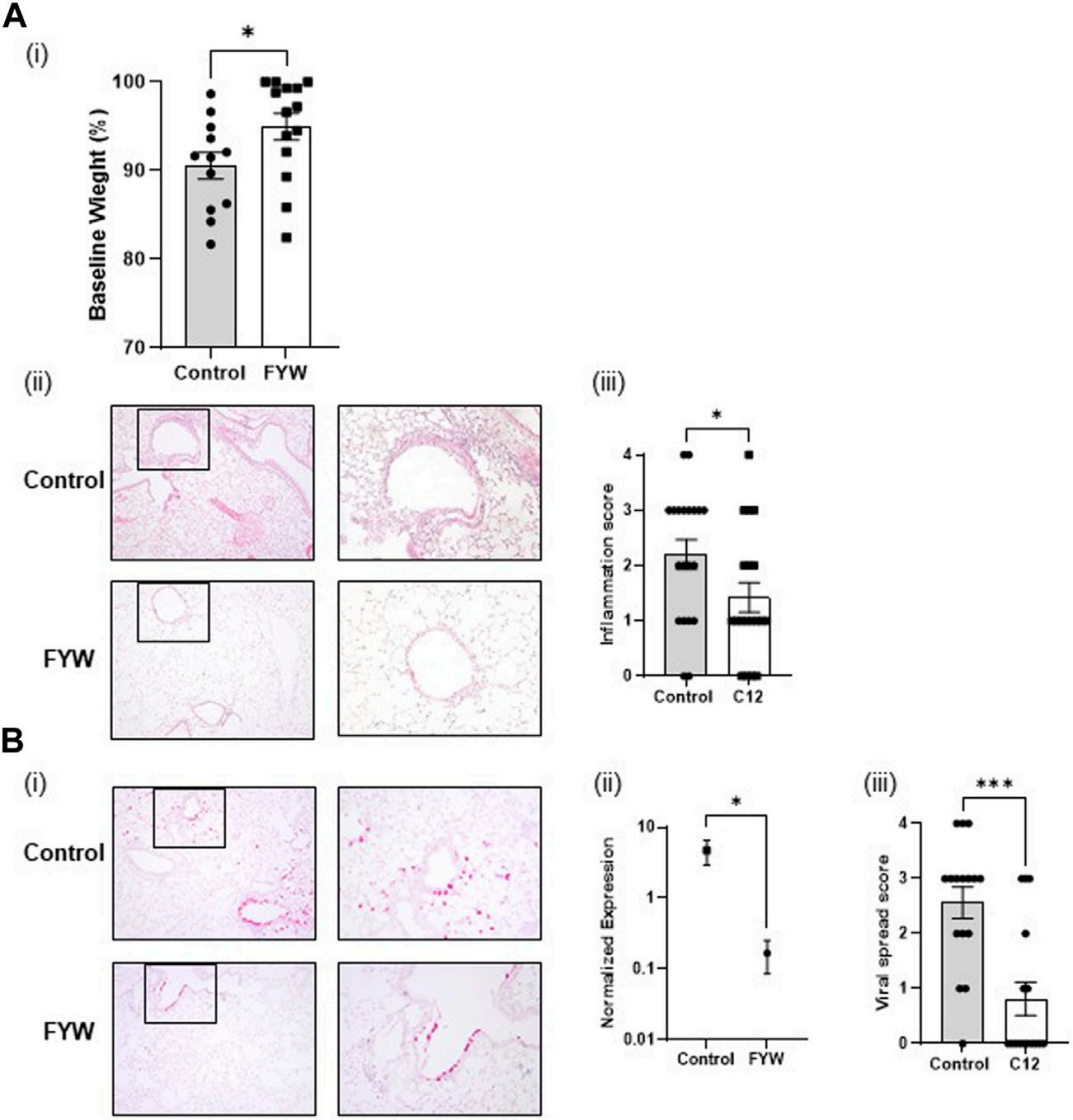
Pooled aromatic amino acid metabolite treatment leads to reduced influenza pathogenesis. **(A)** Weight loss percentage **(i)** in infected adult B6 mice after infection with influenza following 2 weeks of intranasal priming with pooled aromatic amino acid metabolites. Representative images **(A,B)** of lung cross-sections from mice sacrificed 3 days post-infection in the above conditions. H + E stained sections are shown. Boxed areas on the left are magnified adjacently. For scoring, please refer to Supplementary Figure S5 of Anand et al. (2021) **(ii)** Quantification of the degree of lobes with inflammatory infiltrates in lungs, harvested in the above conditions. **(Bi)** Representative images of RNA *in situ* hybridization for the viral transcript NS1. **(ii)** RNA expression determined by real-time PCR for influenza matrix protein in lungs harvested from mice from the same. **(iii)** Quantification of the degree of lobes with influenza viral spread determined from lung cross-sections stained for influenza NS1 by RNA *in situ* from the same mice. For scoring, please refer to Supplementary Figure S13. Graphs depict average SEM *p < or = 3.05 and ***p < 0.001. Mann–Whitney was used for statistical analysis.

To determine whether the FYW treatment reduces influenza pathogenesis and inflammation *in-vivo*, mice were intranasally exposed to FYW or control for 2 weeks daily and then intranasally infected with influenza. We found that mice treated with FYW had improved weight recovery at day 4 post-infection (Figure 9Ai). The mice were sacrificed at this time, and lungs were analyzed by histology and qRT-PCR. Mice treated with FYW had reduced inflammation by histological grading (Anand et al., 2021) (Figures 9Aii, iii). Furthermore, FYW treated mice had decreased levels of viral transcript (28-fold) by qRT-PCR as well as decreased viral spread to the lung periphery as demonstrated by RNA *in-situ* analysis (Figure 9B; Supplementary Figure S13), suggesting that microbial metabolites significantly improve outcomes during influenza pathogenesis.

## 4 Discussion

The gut microbiota exerts myriad beneficial and detrimental effects on host physiology and response to the environment. Microbial metabolites are key mediators in this process and can impact systemic signaling pathways with far reaching effects. Accordingly, gut dysbiosis is involved in the pathogenesis of several diseases and the host response to pathogens (Vivarelli et al., 2019; Sencio et al., 2021; Wei et al., 2021). Therefore a thorough understanding of how microbial metabolites influence the host, and specifically the host immune system, is necessary.

Our previous work demonstrated that a metabolite of tyrosine, 4-OH-phenylpropionic acid, alters IFN signaling and protects from influenza (Steed et al., 2017). The gut microbiota extensively metabolizes the three aromatic amino acids, tryptophan, tyrosine, and phenylalanine (Liu et al., 2020). 4-OH-phenylpropionic acid is produced in such fashion by human gut *Clostridium* microbes, which similarly metabolize tryptophan and phenylalanine (Dodd et al., 2017). These metabolites’ concentrations can reach or exceed typical drug concentrations (Dodd et al., 2017). Therefore we sought to understand the cell specific effects of these metabolites individually and in combination on IFN signaling and infection.

In this study, we screened 17 aromatic amino acid metabolites produced in the human gut by the commensal gut microenvironment. Due to limited and incomplete data evaluating baseline serum concentrations of these metabolites, we studied the metabolites’ effects at an increase of 100 μM in cell culture and at a 1:1 ratio. Baseline concentrations of these metabolites are likely influenced by an individual’s microbiota and host metabolism and therefore difficult to extrapolate for initial experimental investigation (Roager and Licht, 2018). While these final concentrations and ratios may not be physiologic or immediately generalizable, we chose these initial conditions given the potential for therapeutic benefit of metabolite treatment or microbiota alteration.

We found that tryptophan metabolites dramatically reduced IFN signaling. Prior studies have corroborated this finding and demonstrated reduced inflammation and proinflammatory cytokine production in response to tryptophan and its metabolites in bone marrow derived macrophages (Wlodarska et al., 2017; Krishnan et al., 2018; Duanmu et al., 2021). Importantly, we found that the individual metabolites had varied impacts on IFN signaling depending on cell type. Our prior work identified 4-OH-phenylpropionic acid, as an enhancer of IFN signaling in a fibroblast screen (Steed et al., 2017). Here we demonstrate that these metabolites have a differential impact on IFN signaling in epithelial cells and monocytes as well as in combination with different stimuli. While 4-OH-phenylpropionic acid individually increased IFN signaling in the presence of influenza in both monocytes and epithelial cells, the majority of microbial metabolites decreased IFN signaling under various conditions. Furthermore, specific metabolites individually elevated IFN signaling in monocytes but led to reduction in IFN signaling in epithelial cells. This finding hints to different molecules or receptors in diverse cell types interacting with the same metabolite and points to the need for further mechanistic studies (Colosimo et al., 2019).

Notably, IFN signaling was potently reduced from baseline and after exposure to different stimuli when cells were treated with all investigated aromatic amino acids pooled together. This finding was consistent in both cell types investigated, albeit more strikingly in epithelial cells. We found that the main driver was the tryptophan family of metabolites, although phenylalanine and tyrosine metabolites acted synergistically to reduce IFN signaling further. These findings further highlights the impact of these aromatic amino acid metabolites in pooled composition. Although no other study has utilized our pooled aromatic amino acid composition, a previous study have shown that dietary supplementation of aromatic amino acids exert anti-inflammatory effects in a LPS inflammation-induced pig model (Duanmu et al., 2021). Similarly, other studies have also shown reduced inflammatory cytokines such as NF-κB, IL-8, and TNFα in response to aromatic amino acid metabolites (Bansal et al., 2010; Krishnan et al., 2018). While our study did not investigate the capacity of specific bacteria capable of producing these metabolites or *in vitro* bacterially derived metabolites, prior work has demonstrated their immunomodulatory effect *in vivo* (Elsden et al., 1976; Smith and Macfarlane, 1996; Danaceau et al., 2003; Russell et al., 2013; Dodd et al., 2017; Steed et al., 2017; Liu et al., 2020). Further work will undoubtedly focus on understanding how microbial communities and their summed metabolites alter the host-pathogen response.

**FIGURE 10:**
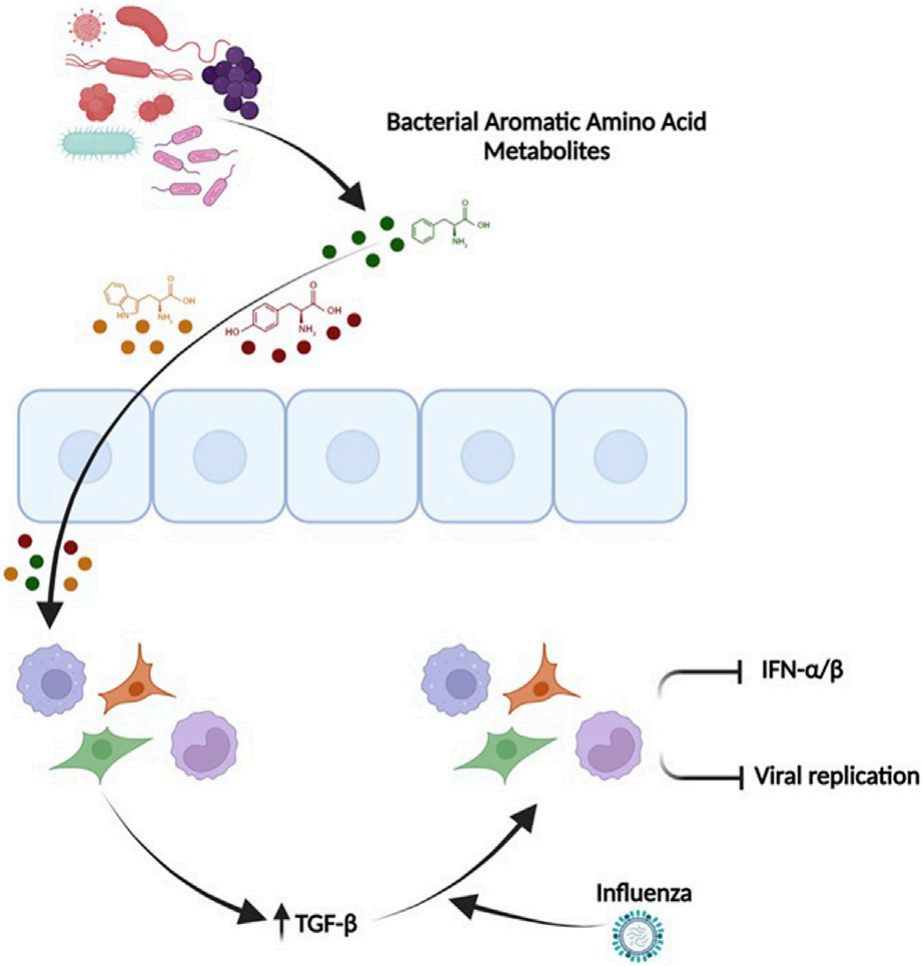
TGF-β mediates the ability of the pooled aromatic amino acid metabolites to decrease IFN signaling and influenza pathogenesis. Microbial aromatic amino acid metabolites are depicted in circles (Green-Phenylalanine, Red-tyrosine, Yellow-Tryptophan). These metabolites affect the cells and potentially enter the cells leading to unique transcriptome with elevated TGFβ. Elevated TGFβ leads to an anti-viral primed state that has reduced IFN signaling, potentially lesser inflammation, and decreased viral recovery. This figure was made in Biorender (http://www.biorender.com/).

Our transcriptomic data confirmed our cell reporter assays by demonstrating a reduction in IFN-α/β signature pathways. The most notable elevations in gene transcription were observed in genes belonging to seemingly different pathways (*SPOCK1*, *EGR2*, *IL31RA* and *ATP6Vd02*). Upon further review, we hypothesized that TGF-beta was the nodal mediator linking these transcripts. TGF-beta leads to elevation of EGR2 (Fang et al., 2011), Spock 1 (Fan et al., 2016) and IL31RA (Jawa et al., 2008; Ferretti et al., 2017). The gene ATP6vd02 is induced in response to IL-6 (Chowdhury et al., 2020), and TGF-beta is known to upregulate IL-6 (Woods et al., 2015; Yang et al., 2017), which aids in tissue repair and reduced influenza induced immunopathology (Furuya et al., 2015). Importantly, we found elevated levels of TGF-beta in our RNA sequencing data. Furthermore, TGF-beta also contributes to protection against influenza in allergic airway diseased mice (Carlson et al., 2010; Li et al., 2015; Rich et al., 2020) and has been shown to reduce IFN signaling (Bedke et al., 2012; Furuya et al., 2015; Denney et al., 2018). Interestingly, TGF-beta is also known to be induced upon influenza infection and is dependent on JNK1 and leads to ER stress (Roberson et al., 2012). We hypothesize that these aromatic amino acid microbial metabolites are inducing elevated TGF-beta signaling with a reduction in IFN signaling and concomitant decrease in immunopathology upon viral challenge (modeled in Figure 10).

Type I IFNs are commonly recognized as key antiviral cytokines. However, some reports demonstrate the cytotoxic impacts of excessive IFN signaling in response to influenza (Herold et al., 2008; Davidson et al., 2014; Hogner et al., 2016). The heightened IFN response can also inhibit inflammasome activation, which plays in important role in the immune response to influenza (Guarda et al., 2011; Pang and Iwasaki, 2011). Interestingly, IFNs also increase susceptibility to secondary bacterial infections (Antonelli et al., 2010; O'Connell et al., 2004). *In vivo* studies of the role of type I IFN during influenza infection are conflicting (Garcia-Sastre et al., 1998; Durbin et al., 2000; Price et al., 2000; Koerner et al., 2007; Mordstein et al., 2008; Szretter et al., 2009; Davidson et al., 2014; Steed et al., 2017). The relative timing of IFN signaling upregulation and response during influenza infection may also predict its effects on viral pathogenesis and outcome for the host. Accordingly, many other biological factors, such as viral strain virulence and immune status of the host undoubtedly contribute. Our finding that specific microbial metabolites decrease IFN signaling adds further evidence that the microbiota is a key influencer of host-pathogen interactions and warrants further work to ascertain fully its impact on disease pathogenesis. Finally, this work demonstrates the broader potential application of these metabolites during viral infections. In particular, understanding the cell specific effects of these metabolites and their pooled composite effects on the host during infections will be necessary to harness their full therapeutic potential.

## Data Availability

The data presented in the study are deposited in the NCBI repository, accession number PRJNA1061515.
