# Supplementary figures and images for: Aromatic amino acid metabolites alter interferon signaling and influenza pathogenesis
Source: Front Mol Biosci. 2024 Jan 23;10:1232573. doi: 10.3389/fmolb.2023.1232573 (PMC10844567; doi:10.3389/fmolb.2023.1232573)

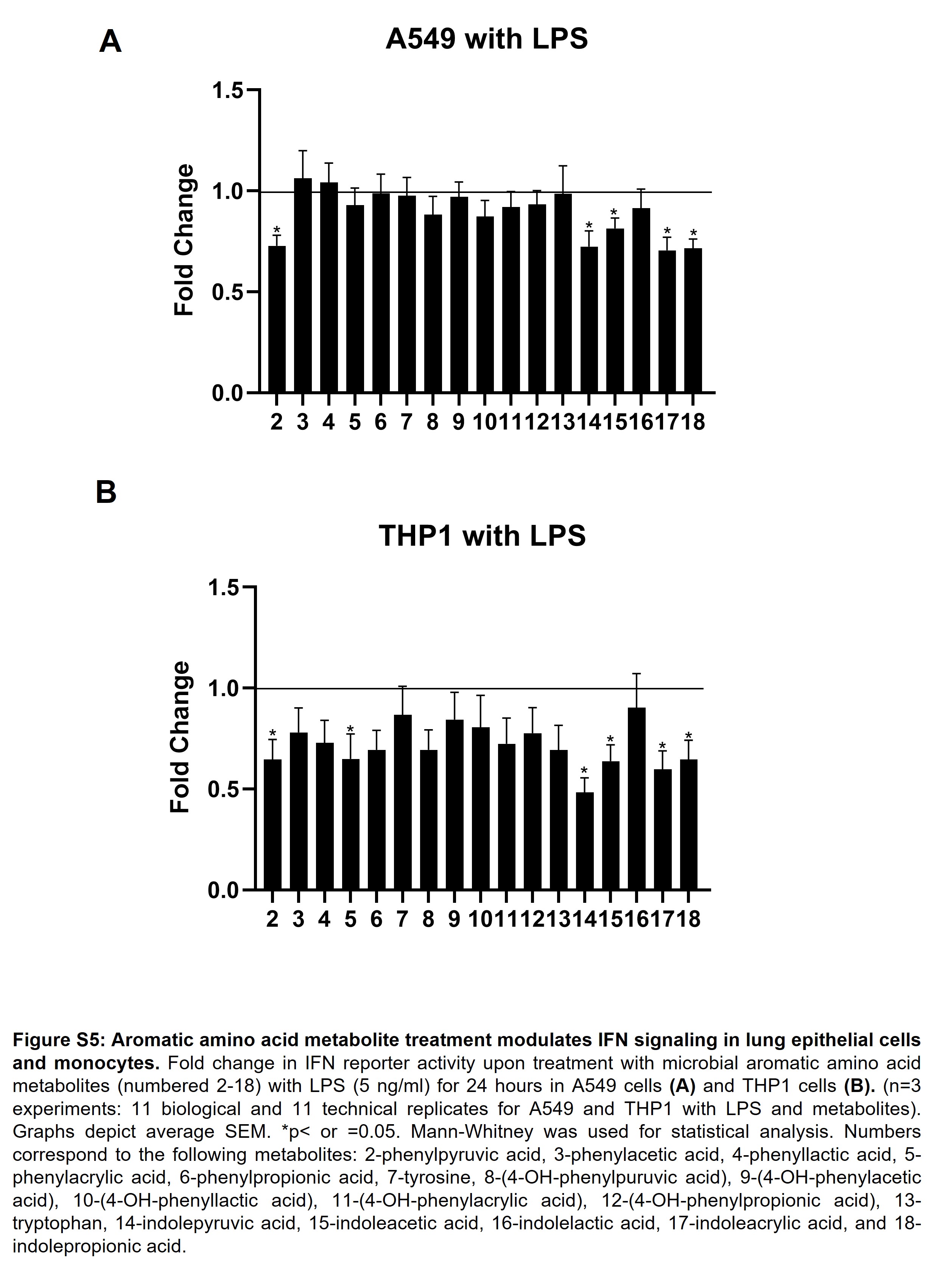

Supplement: Supplementary file 1 [file Image5.jpg]

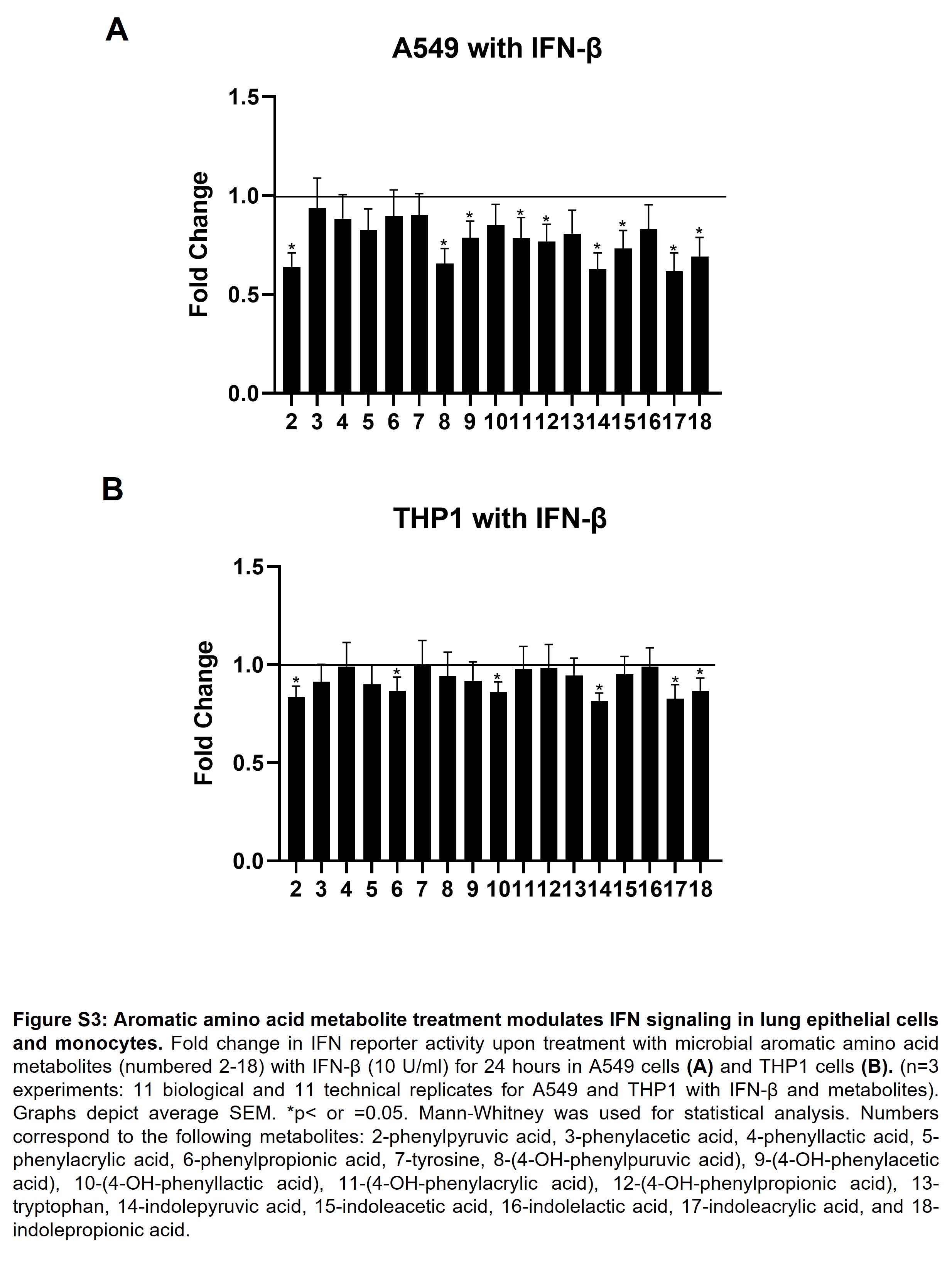

Supplement: Supplementary file 2 [file Image3.jpg]

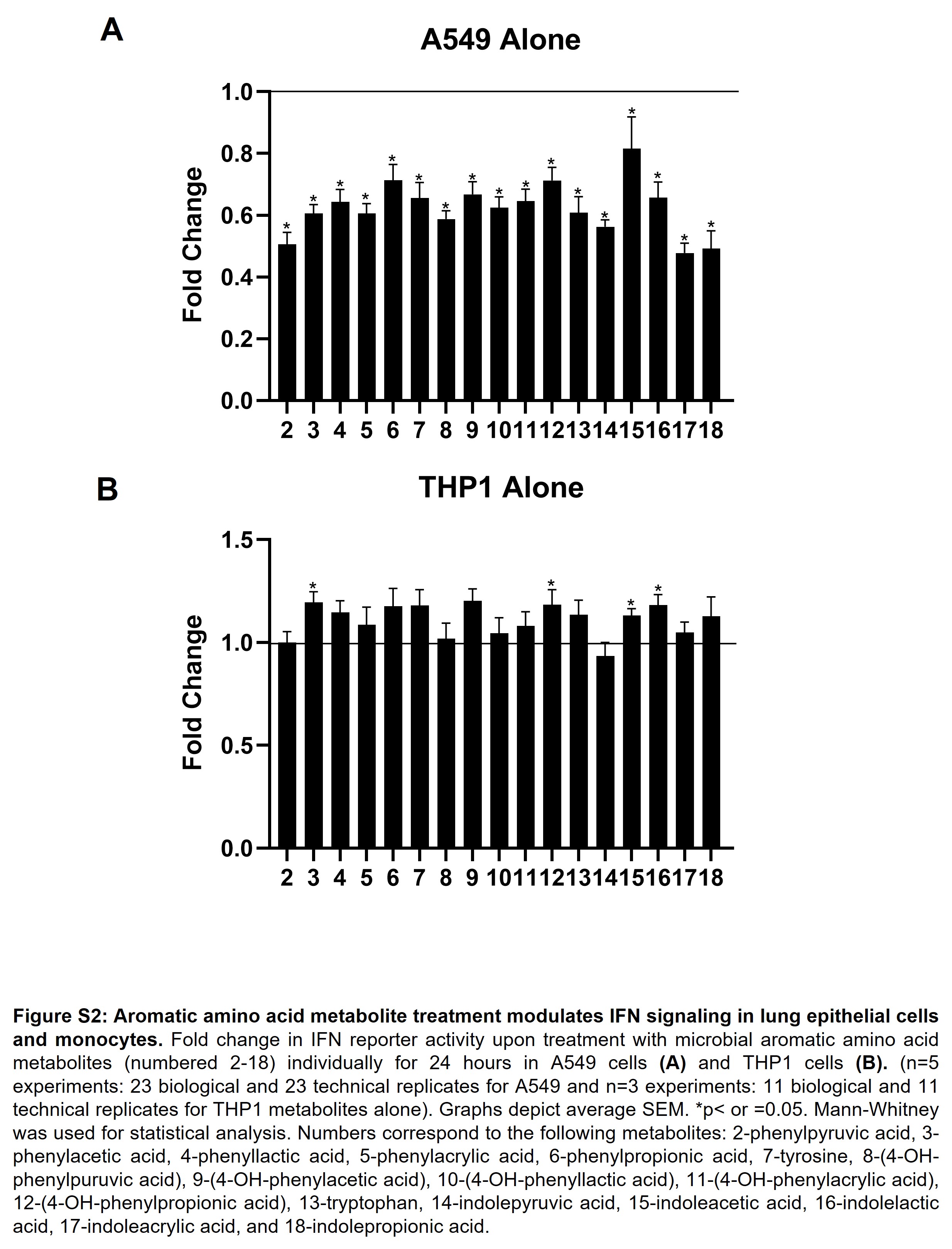

Supplement: Supplementary file 3 [file Image2.jpg]

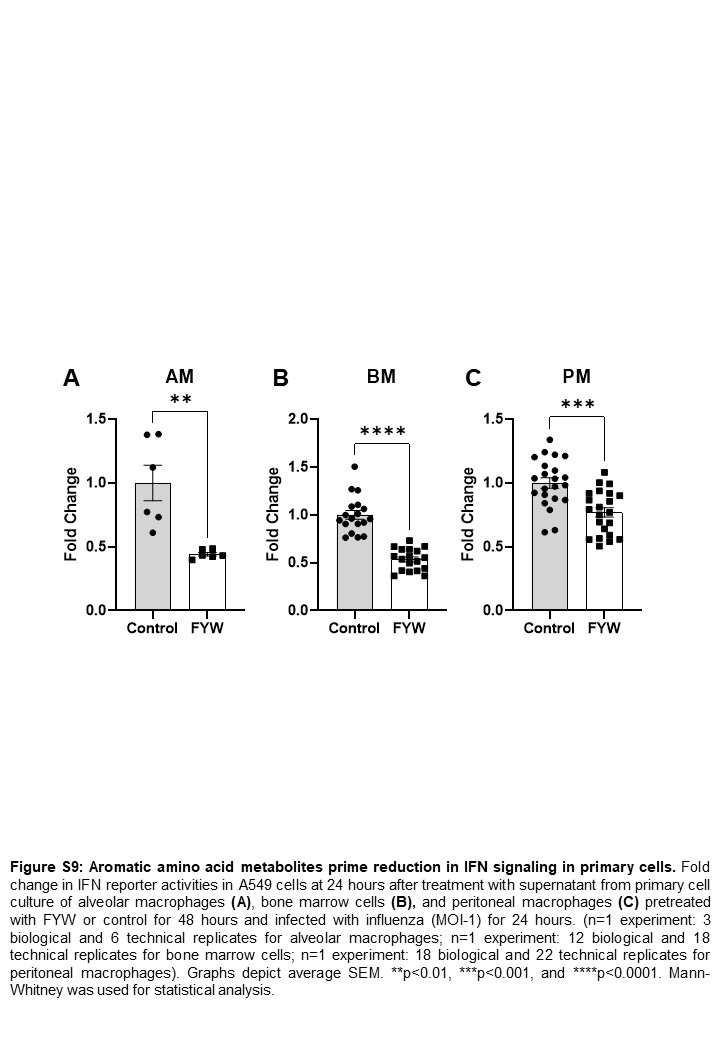

Supplement: Supplementary file 5 [file Image9.JPEG]

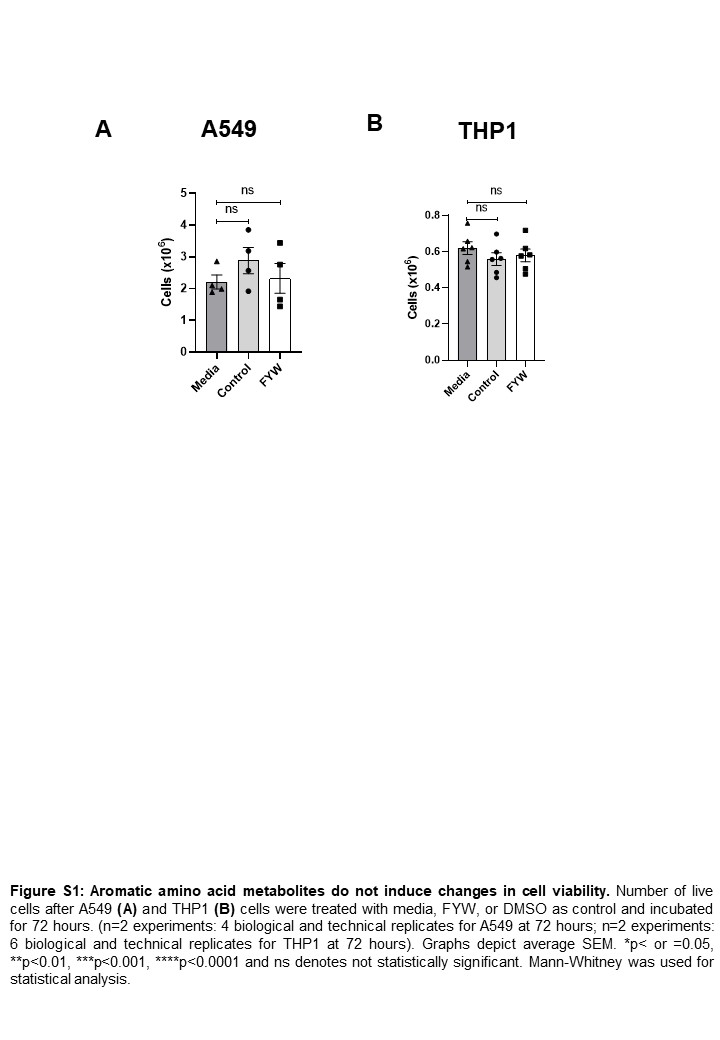

Supplement: Supplementary file 6 [file Image1.JPEG]

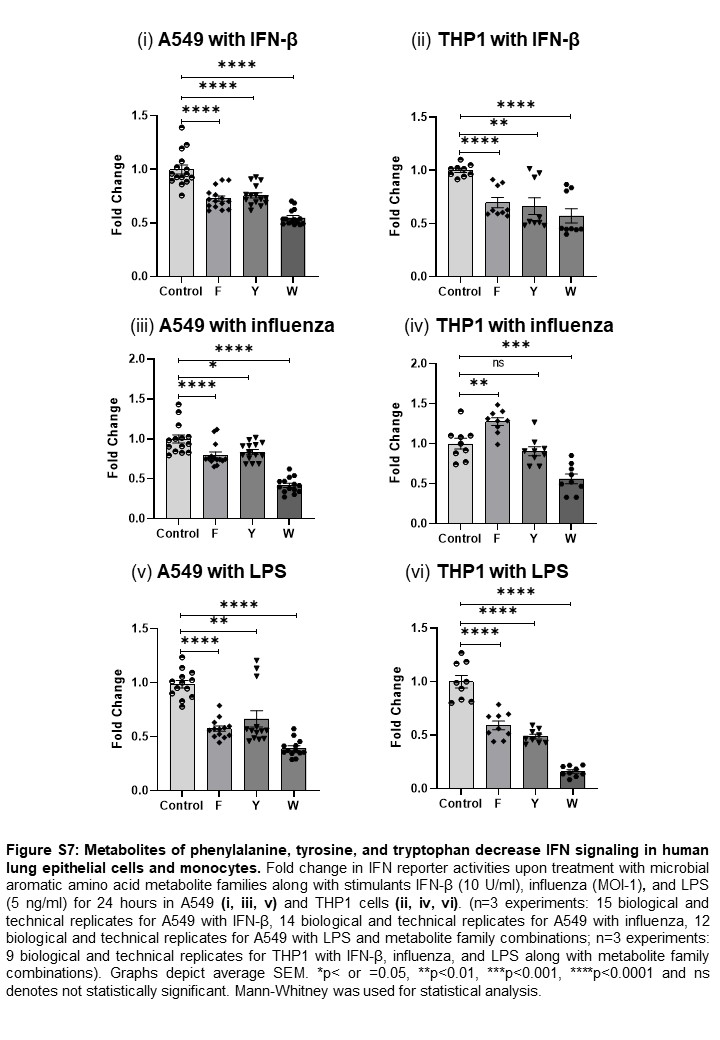

Supplement: Supplementary file 7 [file Image7.JPEG]

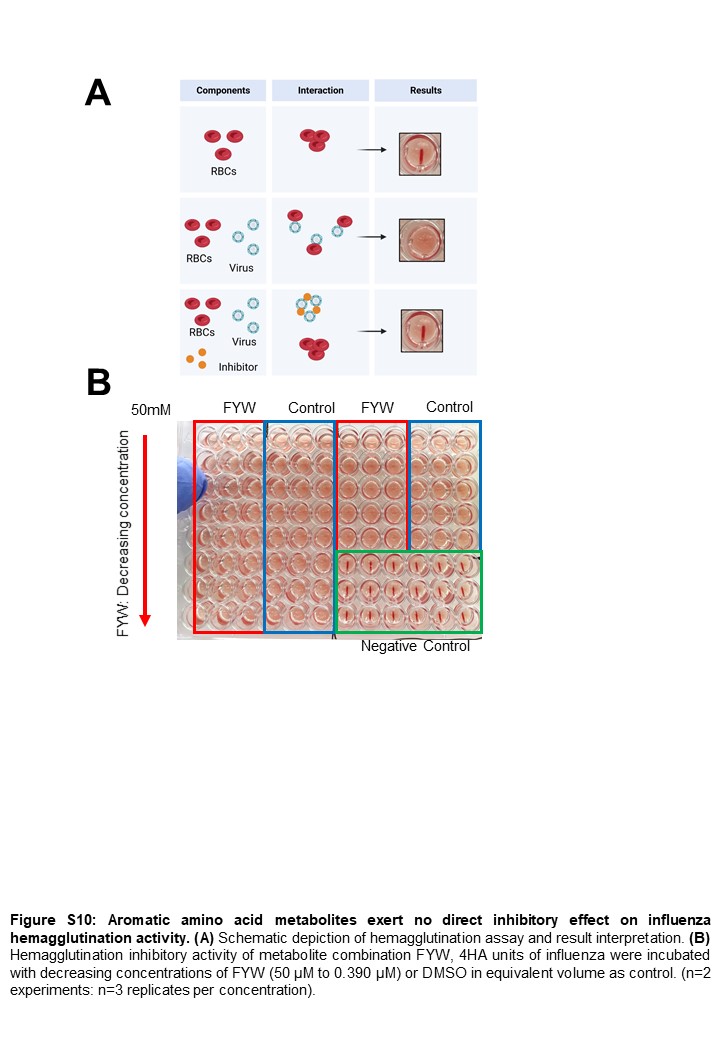

Supplement: Supplementary file 8 [file Image10.JPEG]

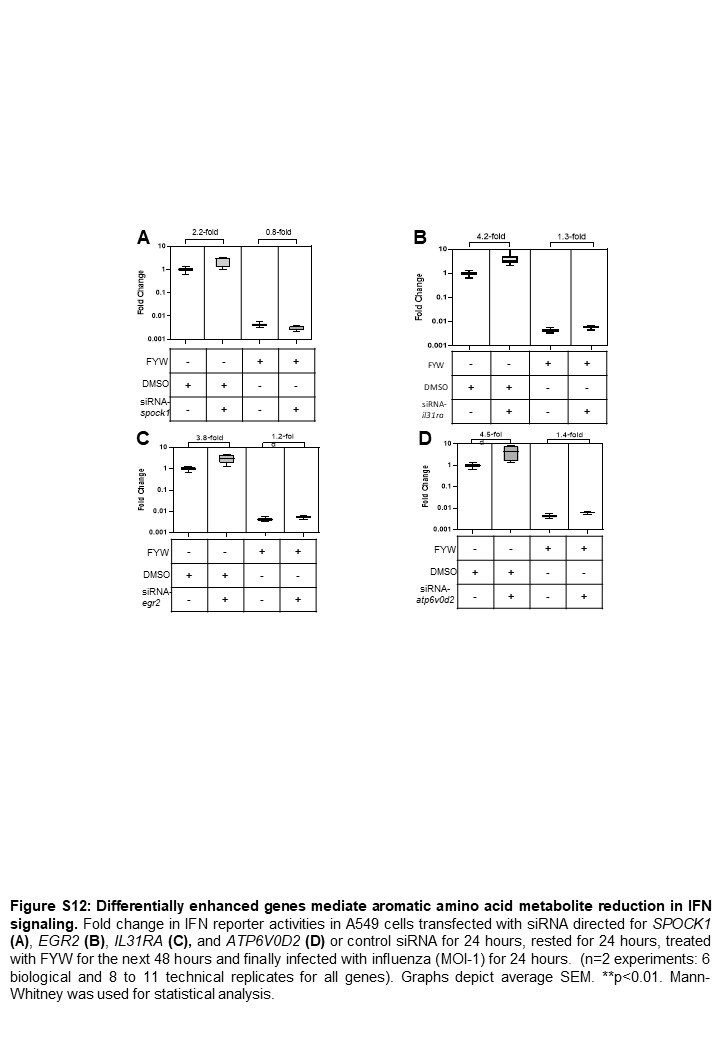

Supplement: Supplementary file 9 [file Image12.JPEG]

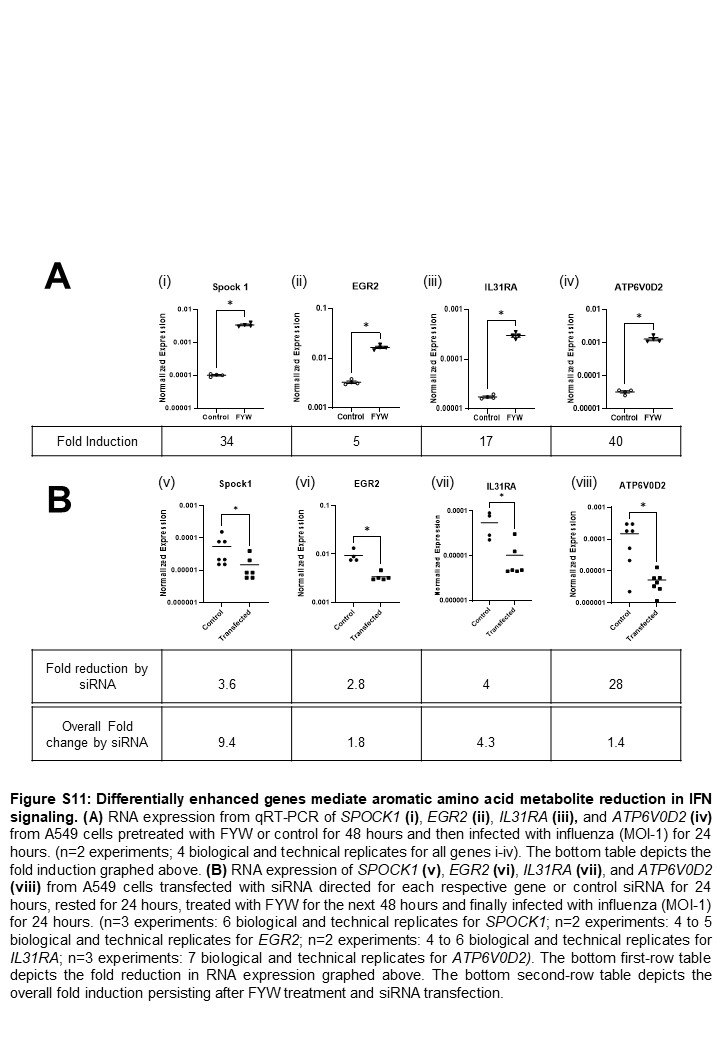

Supplement: Supplementary file 10 [file Image11.JPEG]

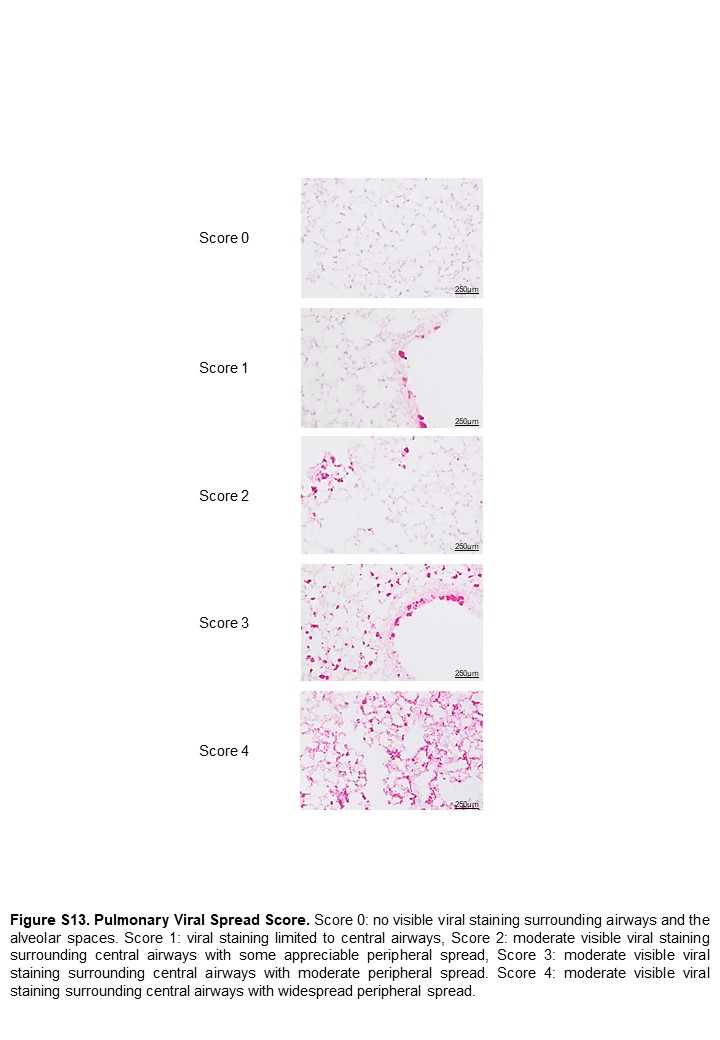

Supplement: Supplementary file 11 [file Image13.JPEG]

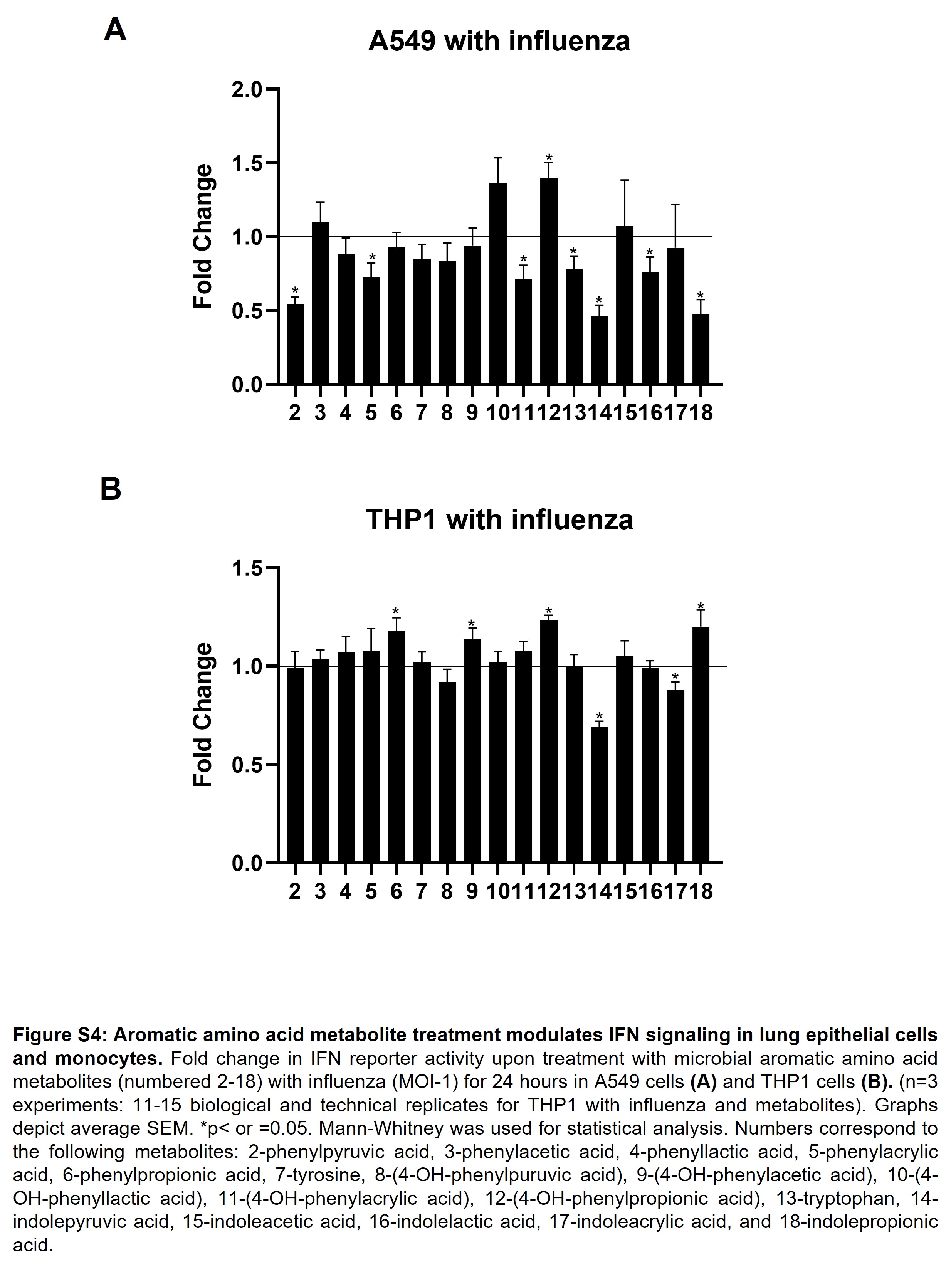

Supplement: Supplementary file 12 [file Image4.jpg]

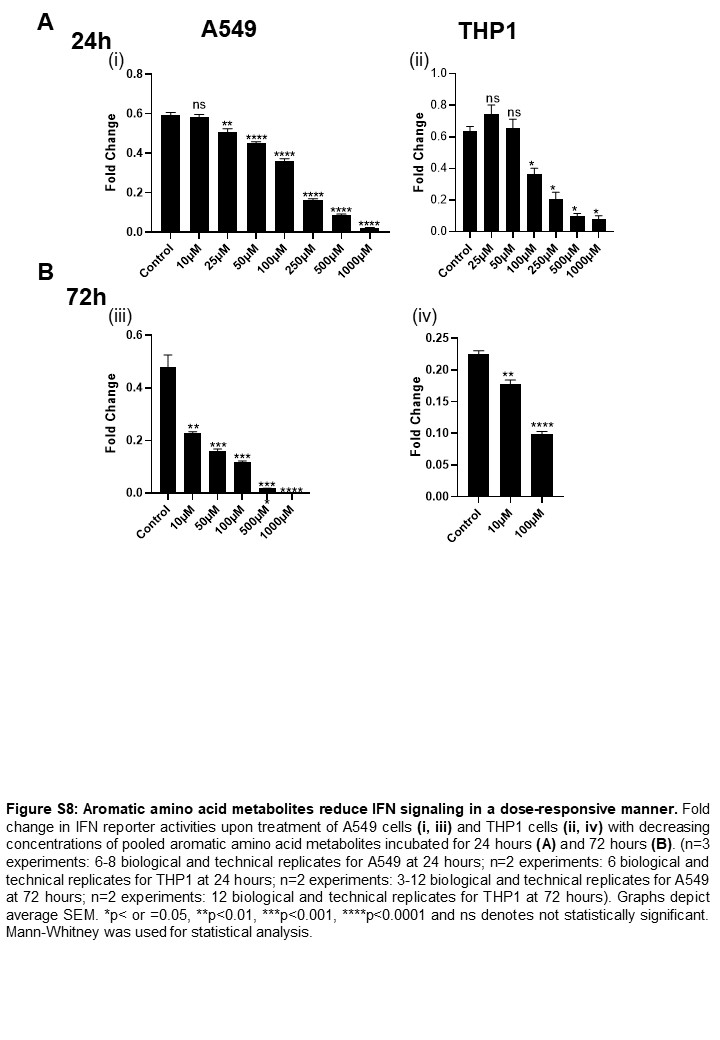

Supplement: Supplementary file 13 [file Image8.JPEG]

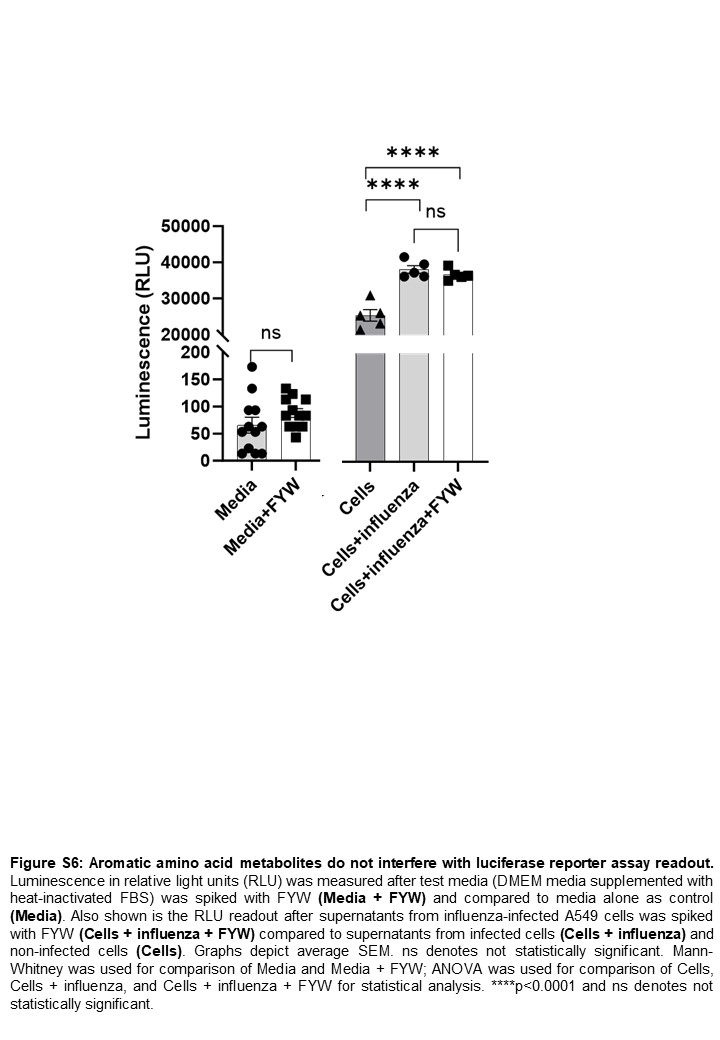

Supplement: Supplementary file 14 [file Image6.JPEG]
