# Supplementary material for: Aromatic amino acid metabolites alter interferon signaling and influenza pathogenesis
Source: Front Mol Biosci. 2024 Jan 23;10:1232573. doi: 10.3389/fmolb.2023.1232573 (PMC10844567; doi:10.3389/fmolb.2023.1232573)
Supplement: Supplementary file 4 [file Table1.docx]

**Supplementary Table 1**: Primers for real-time PCR analysis

| Gene | Sequence (5’-3’) |
| --- | --- |
| Beta-2 microglobulin (β2M) | Forward-TGCTGTCTCCATGTTTGATGTATCT |
|  | Reverse- TCTCTGCTCCCCACCTCTAAGT |
| Influenza (Matrix Protein) | Forward-AAGACCAATCCTGTCACCTCTGA |
|  | Reverse-CAAAGCGTCTACGCTGCAGTCC |
| Spock1 | Forward- CACTGGGTTGGACCTTCGA |
|  | Reverse- CTTTGGTGGCTCAGGCTCT |
| Egr2 | Forward- TCTTCCCAATGATCCCAGACT |
|  | Reverse- TTACGGATTGTAGAGAGTGGAGT |
| IL31RA | Forward- CACACTTCGATTCAGGACAGT |
|  | Reverse- CACATCGCAGAGCTATGACAT |
| ATP6V0D2 | Forward- TCTGATCGAAACGCCATTAGC |
|  | Reverse- CTTCTTTGCTCAATTCAGTGCC |
